# Screening and validation of 3’-Methoxydaidzein as a therapeutic agent in ulcerative colitis based on disulfidptosis-associated molecular clusters

**DOI:** 10.1371/journal.pone.0324586

**Published:** 2025-06-06

**Authors:** Jie Yuan, Chongyong Gao, Wang Xin, Fanlin Meng, Hong Zhang

**Affiliations:** 1 Department of Geriatrics, Chengdu Qingbaijiang District Traditional Chinese Medicine Hospital, Chengdu, China; 2 Emergency Department, Hospital of Chengdu University of Traditional Chinese Medicine, Chengdu, China; 3 Department of Nephrology, Xinqiao Hospital, Army Medical University, Chongqing, China; 4 Department of Geriatrics, Traditional Chinese Medicine Hospital of Meishan, Meishan, China; Jiangsu University, CHINA

## Abstract

**Background:**

Ulcerative colitis (UC) is a recurrent inflammatory condition of the bowel with a multifaceted pathogenesis, including programmed cell death, oxidative stress, and immune-mediated inflammation. As a recently identified type of cell death, disulfidptosis has an unclear role in UC.

**Methods:**

We analyzed clusters of disulfidptosis-related genes (DRGs) and immune cell infiltration in 361 patients with UC from the GSE73661and GSE92415 datasets. Differentially expressed genes (DEGs) were identified using unsupervised clustering methods, and hub genes were selected using machine learning algorithms. Additionally, potential key components of potential traditional Chinese medicines for the treatment of UC were predicted based on hub genes. Finally, experimental validation was performed through qRT-PCR, western blotting, and immunohistochemistry.

**Results:**

We identified two molecular clusters related to disulfidptosis, each showing significant heterogeneity in gene expression and immune profiles. Hub genes associated with disulfidptosis, CXCL1, HMGCS2, AQP8, and SLC26A2, were further screened and validated. Additionally, potential traditional Chinese medicines for UC were predicted. 3’-Methoxydaidzein (MHD), a key constituent of Puerariae Radix, inhibited LPS-induced inflammatory responses in Caco2 cells and alleviated DSS-induced colonic injury in UC mice via upregulation of SLC26A2.

**Conclusion:**

DRGs demonstrate strong discriminatory power in distinguishing UC subtypes. Cluster with high expression of SLC26A2 showed a UC phenotype with a milder degree of damage. Additionally, we identified the hub gene SLC26A2 as playing a significant role in UC, and MHD demonstrates potential as a targeted therapeutic strategy for UC.

## Introduction

Ulcerative colitis (UC) is a persistent and recurrent inflammatory condition affecting the colon and rectum. Since its first discovery in 1859, UC has been recognized as a significant subtype of inflammatory bowel disease (IBD), with its incidence increasing globally over the years [1]. However, its treatment remains challenging despite progress [1,2]. Astonishingly, about 15% of UC patients experienced an aggressive disease course, and even some patients developed colorectal cancer (CRC) [2]. Previous studies have focused on the genetics, microbiome, immune response, environment and intestinal mucosal barrier of UC [3,4]. However, the exact etiology of UC remains incompletely elucidated. Hence, in-depth exploration of the causes and mechanisms of UC is crucial for accurate diagnosis and effective treatment. As reported, various forms of cell death were closely linked to UC [5]. Alongside conventional cell death pathways like apoptosis and necrosis, recent studies on Ferroptosis, Cuproptosis, and other novel forms of cell death have progressively uncovered their potential roles in UC [5–8]. For example, Xu et al. discovered that ferroptosis was involved in intestinal epithelial cell death induced by UC, with NF-κB p65 playing a crucial role in inhibiting ferroptosis [9]. This study suggests that ferroptosis may be a promising therapeutic target in UC. The abnormal activation of these cell death pathways may result in pathological changes, including tissue damage and worsened inflammation [9]. Consequently, modulating cell death pathways has emerged as a critical strategy in UC treatment.

Recent research has revealed that disulfidptosis is a novel form of cell death that occurs due to an excess of disulfide bonds in cellular proteins [10]. Differing from apoptosis and ferroptosis, in glucose-depleted environments, an accumulation of intracellular disulfides occurred in cells expressing high levels of solute carrier family 7 member 11 (SLC7A11) [10]. It is clear that the absence of repair mechanisms leads to disulphide stress and triggers this unique mode of cell death [11]. Recent research have indicated that disulfidptosis is linked to alterations in the cellular redox state and may induce tumor cell death by modifying cytoskeletal protein conformation [12]. Because cancer cells had a robust defense against oxidative stress that heavily relied on the transport of extracellular cysteine, SLC7A11 has been shown to be significantly upregulated in multiple cancer types, such as ovarian, hepatocellular, and colorectal cancers [13]. Despite the crucial role that disulfidptosis plays in immunity and cancer treatment [14,15], studies on the induction of non-tumour cell death by this mechanism are still limited. Identifying appropriate molecular clusters can help provide more specific treatments for UC. Mechanisms in non-tumor contexts have not been thoroughly studied across various disease processes. It is essential to use the expression characteristics of disulfidptosis -related genes to identify UC subtypes.

This research systematically investigated the expression and immune profile of disulfidptosis-related genes (DRGs) in healthy individuals and UC patients. Based on the expression profiles of 25 DRGs, we divided 328 UC patients into two groups and observed differences in immune cell populations. Additionally, two distinct machine learning algorithms were employed to develop a specialized predictive model, revealing distinct molecular clusters among UC patients. Hub genes associated with disulfidptosis, including AQP8, CXCL1, HMGCS2 and SLC26A2 were identified through screening. These hub genes were validated in both the GEO database and our animal experiments. Subsequently, we predicted Traditional Chinese Medicines that might regulate disulfidptosis. We found that 3’-Methoxydaidzein (MHD) significantly alleviated colonic epithelial cell damage both *in vivo* and *in vitro*, suggesting its potential as a treatment for UC.

## Materials and methods

### Data sources

Microarray datasets for UC patients were obtained from the GEO database (www.ncbi.nlm.nih.gov/geo). The datasets include two experimental group datasets (GSE73661 and GSE92415), and a validation group dataset (GSE179285 and GSE206171) [16–18]. Batch effects between GSE73661 and GSE92415 were adjusted with the combat algorithm from the R package “sva”. The datasets were summarized in Table 1. Additionally, DRGs were obtained from previous literature, and the genes were listed in S1 Table [19,20].

**Table 1:** Details of microarray data.

GEO Number	Platform	Disease Samples	Control Samples
GSE73661	GPL6244	166	12
GSE92415	GPL13158	162	21
GSE179285	GPL6480	55	23
GSE206171	GPL19211	114	38

### Differential expression analysis

Differential expression analysis was conducted on the log-transformed dataset with the Limma R package [21]. |log2 fold change| ≥ 1.5 with a p-value < 0.05 was defined as significantly different. The GEO dataset was processed with R version 4.2.2.

### Immune infiltration analysis

The gene expression data matrix was analyzed using the CIBERSORT algorithm to calculate scores for the 22 immune infiltrating cells in each sample [22]. and the results were visualized using the “ggplot2” package.

### GeneMANIA analysis

The protein-protein interaction network of DRGs was constructed by GeneMANIA (http://www.genemania.org).

### Consensus unsupervised clustering of UC patients

“ConsensusClusterPlus” [23] was utilized for cluster analysis, incorporating agglomerative PAM clustering with a Pearson correlation metric (distance = 1) and sampling 80% of the data across 10 iterations. The empirical cumulative distribution function plot was applied to determine the optimal cluster count.

### Weighted gene co-expression network analysis (WGCNA)

Gene expression data were analyzed to determine the median absolute deviation for each gene, excluding the 50% with the lowest MAD values. The “good Samples Genes” function in the WGCNA package was applied to filter outliers among genes and samples [24], and the package was subsequently used to develop a scale-free co-expression network. For module refinement, eigengene dissimilarity was measured, a dendrogram cut-off threshold was set, and overlapping modules were combined. Modules exhibiting a dissimilarity below 0.25 were further unified, yielding seven distinct co-expression modules.

### Functional enrichment analysis

Functional enrichment analyses, including KEGG and Gene Ontology (GO) enrichment, were performed using the “clusterProfiler”. p-values of less than 0.05 were significantly different.

### Using machine learning methods to build predictive models

Using the glmnet R package [25], we implemented lasso-cox regression, employing 10-fold cross-validation to refine the model. The SVM-RFE method, facilitated by the e1071 package t (https://github.com/johncolby/SVM-RFE). Genes identified by both techniques as significant were designated key genes and represented through a Venn diagram.

### Prediction of potential therapeutic traditional Chinese medicine and its active ingredients

Significant hub genes were entered into the Coremine Medical database [26] (https://www.coremine.com/medical/) to identify potential intervention Traditional Chinese Medicines (TCMs) for intervention, using a screening criterion of p* *< 0.05 (S2 Table). If too many drugs were identified, further selection was based on the principles of traditional Chinese medicine theory and clinical usage. The compounds were then screened for oral bioavailability (OB) greater than 30% and drug-likelihood (DL) greater than 0.18 (S3 Table), based on the TCMSP database (https://old.tcmsp-e.com), as previously described [27].

### Animal models

C57BL/6 mice (male), aged eight weeks, were provided by Beijing Huafukang Biotechnology. The mice were randomly assigned to four groups, with 6 mice per group. To construct a model of dextran sodium sulfate (DSS, Aladdin, Shanghai, China) induced UC, 3% (w/v) dextran sodium sulfate (DSS, Aladdin, Shanghai, China) was added to drinking water for 7 consecutive days. Meanwhile, 3’-Methoxydaidzein (MHD) was administered orally at a low dose of 25 mg/kg (MHD-L), a medium dose of 50 mg/kg (MHD-M), and a high dose of 100 mg/kg (MHD-H) in the respective treatment groups for 10 days, while sulfasalazine (SASP, Aladdin, Shanghai, China) [28] at a dose of 370 mg/kg was administered in the positive control group. All mice were euthanized on the 11th day. Blood samples were collected, and colons were excised and measured for length, and then stored at -80°C for future use.

Throughout the study, mice were housed in standard conditions with a 12-hour light/dark cycle, controlled temperature and humidity, and free access to food and water. Health and behavior were monitored at least twice daily. Mice were monitored daily for weight loss, activity level, posture, and signs of distress. Humane endpoints were established to minimize suffering. Mice were euthanized if they exhibited severe distress, including significant weight loss, severe lethargy, hunched posture, rectal prolapse, or inability to access food and water. Euthanasia was performed via CO₂ inhalation followed by cervical dislocation, ensuring a rapid and humane procedure. Once humane endpoint criteria were met, euthanasia was carried out within two hours. No animals died before reaching the humane endpoint. All animal experiments were approved by the Animal Experimentation Ethics Committee of Army Medical University (AMUWEC20224500).

### Toxicity assessment

C57BL/6J mice were administered with MHD (50 mg/kg) by oral gavage for 11 consecutive days. After euthanasia, tissues were collected, and blood samples were obtained for hepatic and renal function analysis. Major organs were harvested for hematoxylin and eosin (HE) staining.

### Cell culture and treatment

Human intestinal epithelial cells (Caco2) were obtained from the American Tissue Culture Conservation Center (Manassas, USA) and cultured in DMEM 1X medium containing 10% fetal bovine serum (Meron Bio, Dalian, China). Cells were then exposed to LPS (1 or 10 μg/ml) or different concentrations of Formononetin (FMN, Aladdin, Shanghai, China), Beta-sitosterol (BST, Aladdin), 3’-Methoxydaidzein (MHD, Aladdin) and Daidzein-4,7-diglucoside (DDC, MedChemExpress, NJ, USA) for 24 hours. Caco2 cells were transfected with SLC26A2 overexpression plasmids (OE SLC26A2) (Youbio, Hunan, China) by using Lipofectamine 3000 (Invitrogen, Carlsbad, USA).

### Cell viability assay

Caco2 cells were plated in 96-well plates, and treated with different concentrations of MHD, DDC, FMN and BST for 24 h. Cell toxicity was assessed with the CCK-8 kit (MedChemExpress).

### qRT-PCR

Colon tissues and Caco2 cells were collected, and total RNA was extracted using Trizol. Quantitative real-time PCR (qRT-PCR) was performed using the CFX96 detection system (Bio-Rad, USA). β-actin was used as the internal control, and target gene expression was quantified using the 2^^-ΔΔCT^ method. Primer sequences are provided in S4 Table.

### Western blot

Total protein was extracted from colon tissue. Proteins were separated by SDS-PAGE, transferred to a PVDF membrane, and incubated with 5% skimmed milk for 1 hour. Primary antibodies against SLC26A2 (27759-1-AP, Proteintech, Wuhan, China) and β-actin (AF0003, Beyotime, Shanghai China) were incubated overnight at 4°C. Subsequently, the membranes were incubated with HRP-labelled secondary antibodies for 1 hour. Signals were detected using an enhanced chemiluminescence method. ImageJ software was used for grayscale analysis.

### Calcein-AM/PI staining

The treated Caco2 cells were stained using the Calcein-AM/PI kit (MA0361, Melun Bio, Dalian, China).

### HE and immunohistochemical staining

Mouse tissue sections were stained with H&E to assess damage to individual tissues. Immunostaining was carried out as described previously [29]. Sections were incubated overnight at 4°C with the SLC26A2 primary antibody, then treated with a secondary antibody and stained using 3,3’-diaminobenzidine.

### Statistical analysis

Statistical analysis was performed using Prism (GraphPad Software, version 8.0). All data were presented as mean ± SD. Statistical tests included unpaired t-tests and one-way ANOVA. *p* < 0.05 was considered statistically significant.

## Results

### Molecular characteristics and immune infiltration assessment of DRGs in UC patients

**Fig 1:**
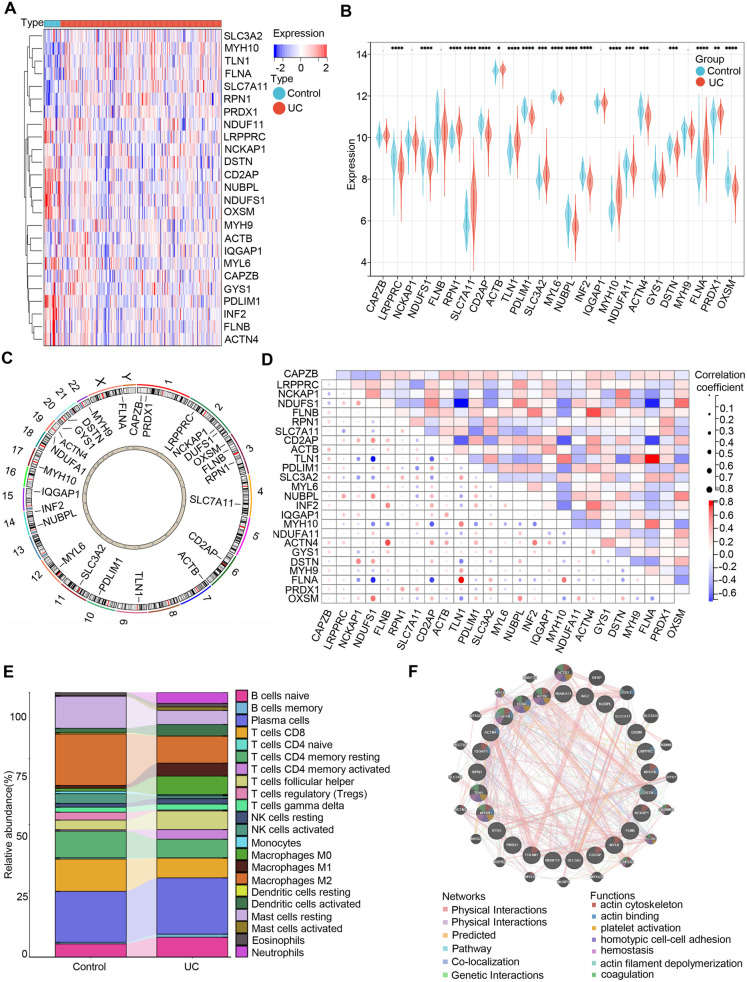
Molecular characteristics and immune infiltration assessment of DRGs in UC patients. (A) Heatmap of Differential Expression of DRGs in Control and UC Patients. (B) Box plots showing the expression of 25 DRGs between UC and control groups, * *p* < 0.05, ** *p* < 0.01, *** *p* < 0.001, **** *p* < 0.0001. (C) The chromosomal locations of the 25 DRGs. (D) Correlation analysis of the 25 DRGs using a dot plot. (E) The abundance of 22 immune cell infiltrations between UC patients and the control group. (F) Network diagram of the relationships among the 25 DRGs.

To reveal the role of disulfidptosis in the pathogenesis of UC, we examined GEO RNA-seq data from 328 UC patient samples and 33 samples from a colon control group (S5 Table). The analysis workflow was depicted in S1 Fig. We identified 25 DRGs, and the visualized heatmap illustrated distinct DRGs expression patterns between the control and UC groups (Fig 1A). Elevated expression levels of RPN1, SLC7A11, SLC3A2, among others, were observed in UC patients and validated in colonic tissues of DSS-induced UC mice (Fig 1B and S2 Fig). Then, the chromosomal locations of the 25 DRGs are then shown (Fig 1C). Spearman correlation analysis identified significant interactions between specific mitochondrial regulatory genes (such as NUBPL and LRPPRC). Conversely, some antagonisms were noted between FLNB and SLC7A11 (Fig 1D). Further, immune infiltration analysis revealed that the levels of M0 Macrophages, Mast cells activated, NK cells resting, and Plasma cells were relatively higher in UC patients, (Fig 1E), implying a close correlation between UC onset and the immune system. Additionally, the circular gene plot excellently exhibited the regulatory networks and functions of DRGs (Fig 1F). These findings indicate that DRGs are pivotal in regulating immune cell infiltration and molecular processes in UC.

### Identification of clusters in UC and immune cell infiltration features

**Fig 2:**
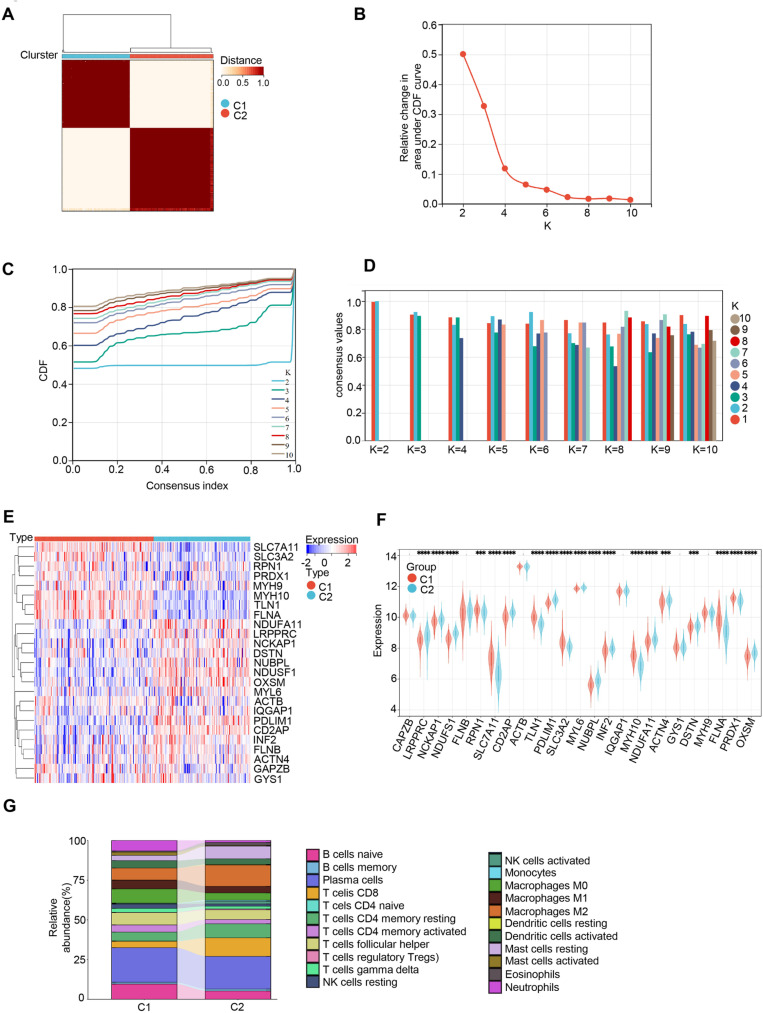
Identification of UC clusters and comparison of immune cell infiltration characteristics. (A) Based on the expression of DRGs, UC patients’ samples were divided into two clusters using consensus clustering algorithm (k = 2). (B) Scores for consensus clustering. (C) CDF delta area curves. (D) Heatmap of non-negative matrix. (E) Heatmap of DRG expression between Cluster 1 and Cluster 2. (F) Expression of 25 DRGs between Cluster 1 and Cluster 2, * *p* < 0.05, ** *p* < 0.01, *** *p *< 0.001, **** *p* < 0.0001. (G) Richness of 22 immune cell infiltrations between Cluster 1 and Cluster 2.

An unsupervised clustering algorithm was employed to group 328 UC samples based on the 25 DRGs, aiming to identify disulfidptosis-related expression patterns. The results indicated that the clustering was most stable at k = 2 (Fig 2A–D). Subsequently, expression analyses of the 2 Clusters showed that Cluster 1 exhibited higher expression levels of SLC3A2, MYH10, TLN1, FLNA, RPN1, SLC7A11, PRDX1, and others (Fig 2E and F). Further, immune infiltration analysis indicated that Cluster 1 had a larger proportion of Neutrophils and Mast cells activated, while Cluster 2 exhibited increased activation of CD8 + T cells, monocytes, and NK cells (Fig 2G).

### Differential gene expression and weighted gene co-expression network analysis of disulfidptosis -related clusters

**Fig 3:**
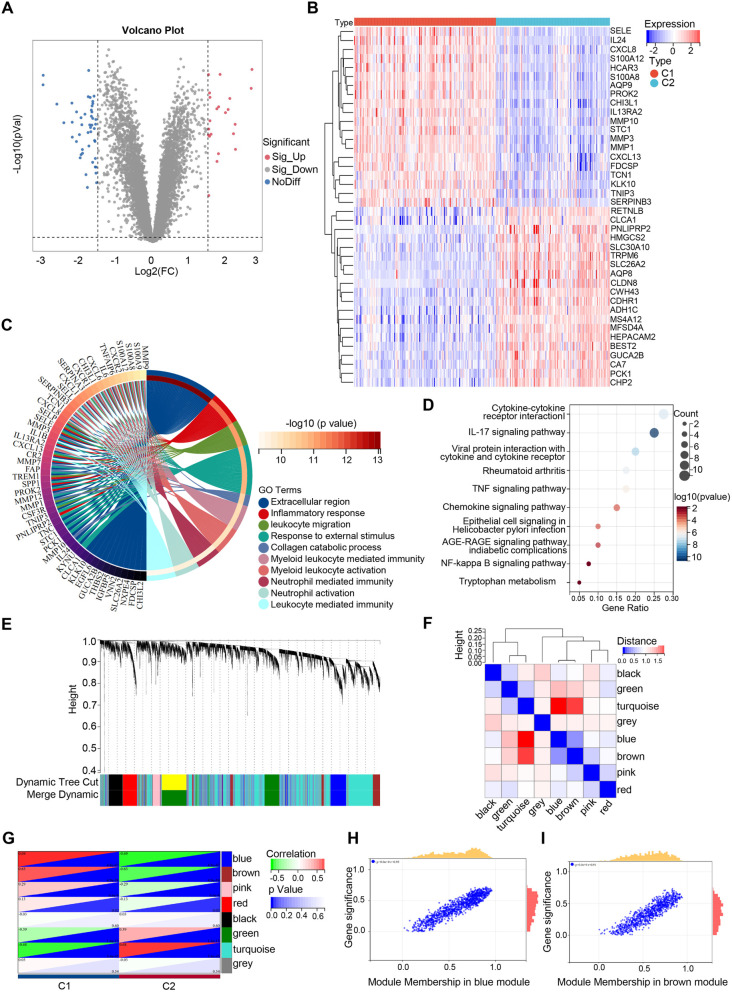
Differential gene expression and weighted gene co-expression network analysis of disulfidptosis-related molecular clusters. (A) Volcano plot of DEGs between the two DRG clusters. (B) Heatmap of the DEGs between the two DRG clusters. (C and D) GO and KEGG enrichment analyses of the DEGs between the two DRG clusters. (E) Dendrogram of co-expressed genes, where different colors represent different gene co-expression modules. (F) Module feature clustering diagram. (G) Module-trait relationships, with each cell listing the corresponding *p*-value and correlation coefficient. (H and I) Correlation scatter plot.

To explore distinct gene expression patterns between Cluster 1 and Cluster 2, 69 differentially expressed genes (DEGs) were identified (Fig 3A). Heatmap visualization highlighted the presence of several significant DEGs (Fig 3B). Subsequently, GO analysis further showed that the altered genes were mainly linked to processes such as leukocyte migration and inflammatory response (Fig 3C). KEGG pathway enrichment analysis revealed that the disulfidptosis-related cluster was mainly associated with pathways such as cytokine-cytokine receptor interaction, TNF signaling, NF-kappa B signaling, AGE-RAGE signaling in diabetic complications, and tryptophan metabolism (Fig 3D). In addition, we analyzed the genes in the disulfidptosis-associated clusters by WGCNA, yielding eight distinct colour modules containing a total of 8580 genes (Fig 3E and F). Among them, the UC cluster exhibited pronounced associations with the blue module (1100 genes) and the brown module (966 genes) (Fig 3G–I).

### Identification of cluster-specific DEGs

**Fig 4:**
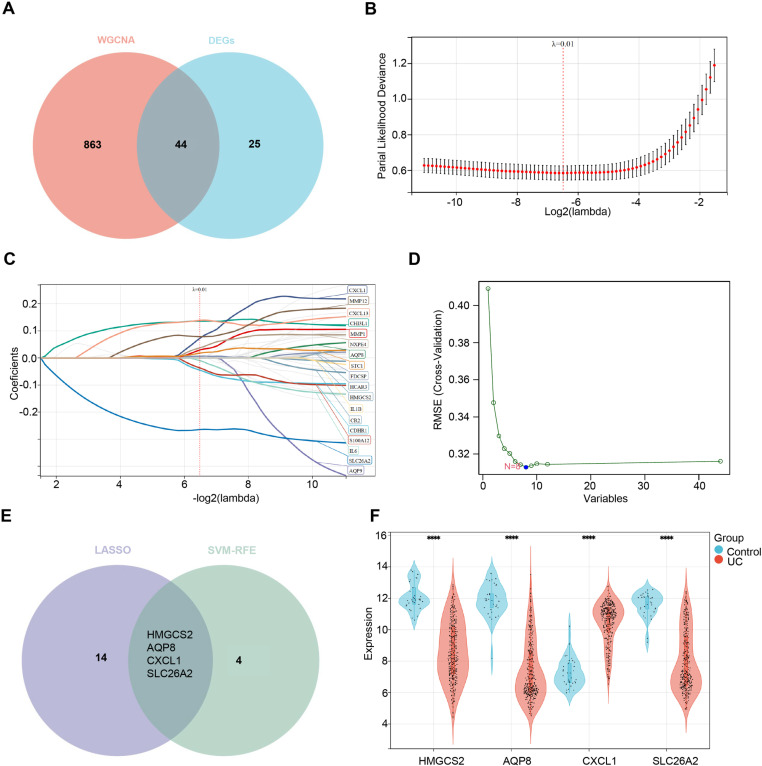
Identification of specific cluster DEGs based on two disulfidptosis-related molecular clusters. (A) Venn diagram of the intersection of DEGs between two clusters and WGCNA hub module genes. (B) Error plot of different lambda values in LASSO. (C) Logarithm of the partial likelihood deviance plotted through Lasso regression in tenfold cross-validation. (D) Error plot of different feature numbers in SVM-RFE. (E) Venn diagram of the key genes from two machine learning methods. (F) Box plots of hub genes expression between UC and control groups (GSE73661 and GSE92415).

In order to reveal potential hub genes within the disulfidptosis-related cluster, our analysis encompassed hub genes from the blue module and brown module within the Cluster WGCNA, along with Cluster DEGs, resulting in a collection of 44 pertinent overlapping genes with distinct DEGs (Fig 4A). Moreover, we delved into the expression profiles of these 44 cluster-specific DRGs, using two machine learning algorithms. The LASSO method selected 18 genes (Fig 4B and C), and the SVM-RFE algorithm identified 8 genes (Fig 4D). Notably, 4 genes were jointly recognized by both algorithms (Fig 4E). Conclusively, we examined the expression of these 4 hub genes, discovering significant upregulation of CXCL1 in UC patients, whereas HMGCS2, AQP8, and SLC26A2 exhibited significant downregulation in UC patients (Fig 4F). In addition, Cluster 1 exhibited higher expressions of CXCL1 and lower expression of HMGCS2, AQP8, and SLC26A2 compared to Cluster 2 in UC (S3A Fig). To further investigate this, we established an *in vitro* UC model with varying degrees of injury to simulate the two subtypes based on clinical data (S6–S9 Tables). We observed that as the severity of cell injury increased, the expression of the hub genes HMGCS2, AQP8, and SLC26A2 significantly decreased, while the expression of CXCL1 increased (S3B–E Fig). These results suggest that Cluster 1 corresponds to the more severely damaged phenotype, whereas Cluster 2 represents the less damaged phenotype in UC.

### Identification of hub genes and prediction of potential therapeutic traditional Chinese medicines

**Fig 5:**
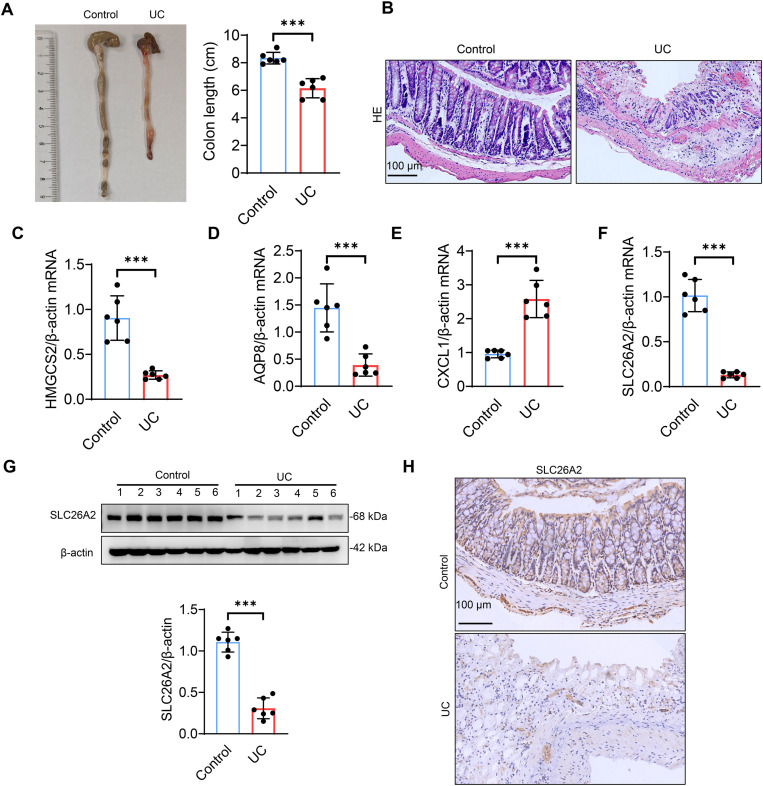
Experimental identification of hub genes. (A) Analysis of colon length in control and UC mice (n = 6). (B) Representative images of HE-staining in colonic tissue from control and UC mice, scale bar = 100μm (n = 6). (C-F) Expression levels of HMGCS2, AQP8, CXCL1 and SLC26A2 were analyzed by qPCR (n = 6). (G) Expression levels of SLC26A2 were analyzed by Western blot (n = 6). (H) Representative immunohistochemical images of SLC26A2, scale bar = 100μm. All data are represented as mean ± SD, * *p* < 0.05, ** *p* < 0.01, *** *p *< 0.001.

**Fig 6:**
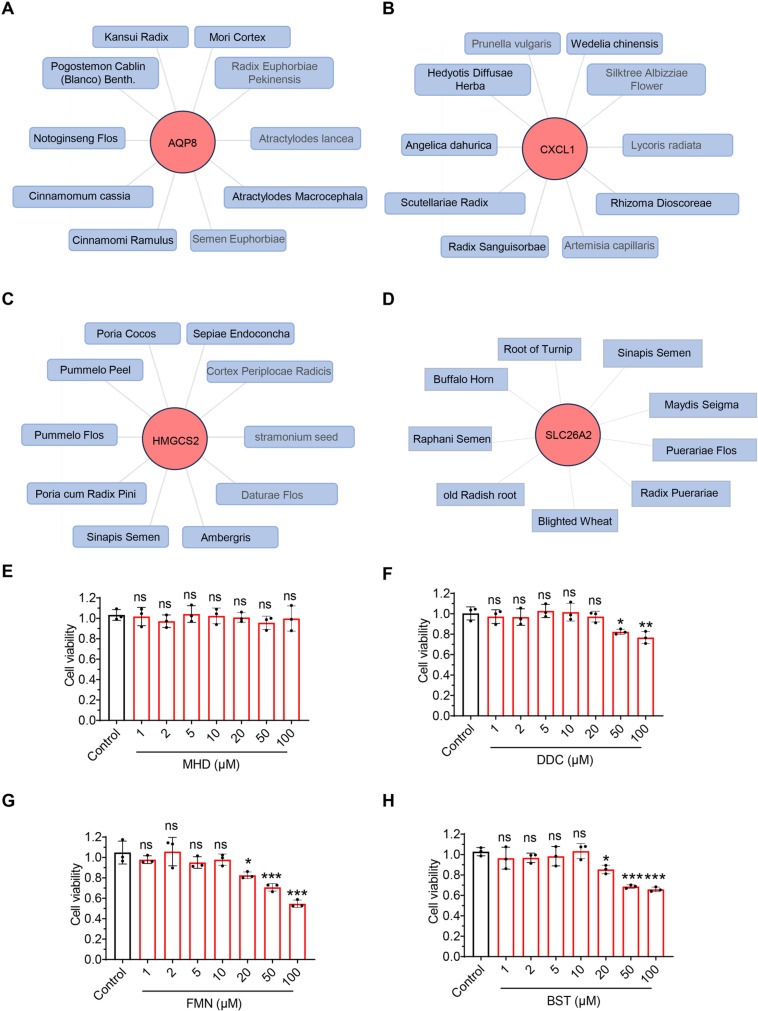
Prediction of potential therapeutic Chinese Medicines based on Hub Genes. (A-D) For AQP8, CXCL1, HMGCS2 and SLC26A2, Chinese medicines with potential intervention effects were mapped using the Coremine Medical database, filtered with a standard of *P* < 0.05. (E) Cell viability assay of Caco2 cells treated with control or different concentrations of MHD, DDC, FMN and BST. n = 3. All data are represented as mean ± SD, ns, not significant, * *p* < 0.05, ** *p* < 0.01, *** *p* < 0.001.

Further, we used external databases for validation. The results revealed that samples from 114 UC patients remained divided into 2 clusters and that Cluster 1 exhibited higher expression levels of SLC3A2, MYH10, FLNA, RPN1, SLC7A11, PRDX1, consistent with the data we analyzed (S4A–E Fig). Notably, the expression of the hub gene further confirms our findings (S4F–J Fig). Subsequently, a DSS-induced UC mouse model was constructed. qPCR analysis demonstrated that SLC26A2 was the most significantly downregulated gene in the colon tissue of UC mice (Fig 5A–F). To further validate this, we performed Western blotting and immunohistochemical staining at the protein level, confirming a marked downregulation of SLC26A2 in the colon tissue of UC mice (Fig 5G and H), aligning with the bioinformatics findings. Additionally, we mapped the hub genes to the Coremine Medical database and obtained potential therapeutic traditional Chinese medicines (Fig 6A–D), including Panax Notoginseng, Cinnamomum cassia, Scutellariae Radix, Poria Cocos, and Radix Puerariae, which have shown certain efficacy in treating UC [30–34]. Finally, based on traditional Chinese medicine theory and previous literature reports, we conducted screening of the above-mentioned Chinese herbs targeting SLC26A2 and found that Puerariae Radix has a significant anti-inflammatory and anti-diarrheal effect [34,35]. Ultimately, we obtained and screened the active components of Puerariae Radix from the TCMSP database, including MHD, DDC, FMN and BST, as potential compounds for the treatment of UC (Table 2).

**Table 2:** Screening of key components of Traditional Chinese Medicine Puerariae Radix.

Name	OB (%)	DL	Chemical Formula
Beta-sitosterol	36.91	0.75	C_29_H_50_O
Daidzein-4,7-diglucoside	47.27	0.67	C_27_H_30_O_14_
3’-Methoxydaidzein	48.57	0.24	C_16_H_12_O_5_
Formononetin	69.67	0.21	C_16_H_12_O_4_

### MHD inhibits LPS-induced inflammatory response in Caco2 cells through upregulation of SLC26A2

**Fig 7:**
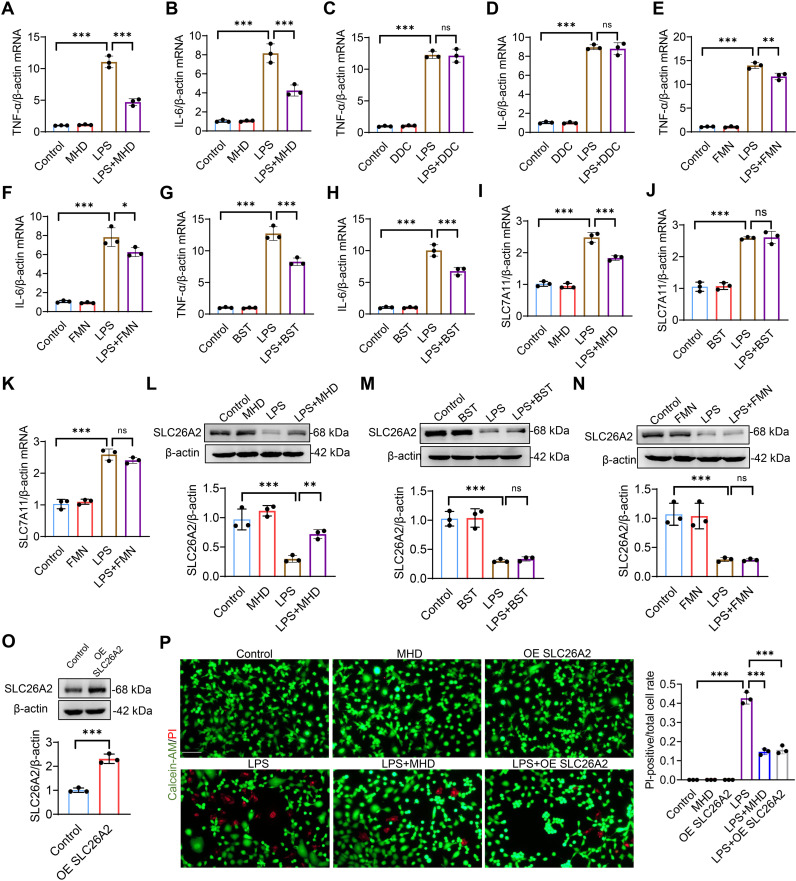
Protective effects of the main components of Puerariae Radix on LPS-induced colonic epithelial cells. (A-H) Caco2 cells were treated with LPS (10 μg/ml) or 3’-Methoxydaidzein (MHD, 10 μM), Daidzein-4,7-diglucoside (DDC, 10 μM), Formononetin (FMN, 10 μM) and Beta-sitosterol (BST, 10 μM) for 24 hours, then the relative expression levels of TNF-α and IL-6 were analyzed by qPCR, (n = 3). (I-K) The expression of SLC7A11 was analyzed by qPCR, (n = 3). (L-O) The expression of SLC26A2 was analyzed by Western blot, (n = 3). (P) Representative images of Calcein-AM/PI staining of Caco2 cells, scale bar = 100 μm, (n = 3). All data are represented as mean ± SD, ns, not significant, * *p* < 0.05, ** *p* < 0.01, *** *p *< 0.001.

To further validate the role of Puerariae Radix active ingredients in UC, we investigated the protective effects of Puerariae Radix active ingredients against intestinal injury under LPS-induced inflammatory conditions using Caco2 cells as a cell model. Cell activity assays showed that MHD, DDC, FMN and BST were not toxic to Caco2 cells at concentrations ≤ 10 μM (Fig 6E–H). We subsequently found that MHD, FMN and BST all significantly reduced LPS-induced upregulation of inflammatory factors TNF-α and IL-6 levels in Caco2 cells, while DDC showed no effect (Fig 7A–H). Meanwhile, we found that MHD significantly reduced the LPS-induced elevation of SLC7A11 expression in Caco2 cells (Fig 7I–K). Further, the western blot showed that only MHD was able to restore the LPS-induced decrease in SLC26A2 expression in Caco2 cells, and both SLC26A2 overexpression and that MHD treatment rescued the LPS-induced decrease in Caco2 cell viability (Fig 7L–P). These results suggest that MHD inhibits LPS-induced inflammatory response and cell death in Caco2 cells by upregulating SLC26A2.

### MHD ameliorates DSS-induced UC

**Fig 8:**
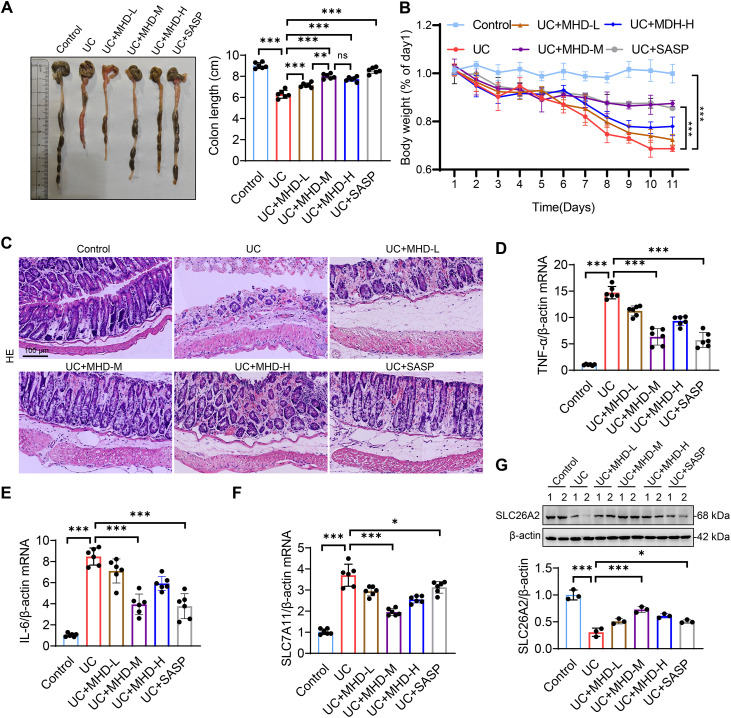
Protective effect of MHD against DSS-induced UC. (A) Analysis of colon length in control and UC mice treated with MHD (25, 50 and 100 mg/kg), SASP (370mg/kg) or saline (n = 6). (B) Measurement of mouse body weight. (C) Representative images of HE staining of murine colon tissue, scale bar = 100μm. (D-F) The expression levels of TNF-α, IL-6 and SLC7A11 were analyzed by qPCR (n = 6). (G) The expression of SLC26A2 was analyzed by Western blot (n = 3). All data are represented as mean ± SD, ns, not significant, * *p* < 0.05, ** *p* < 0.01, *** *p *< 0.001.

**Fig 9:**
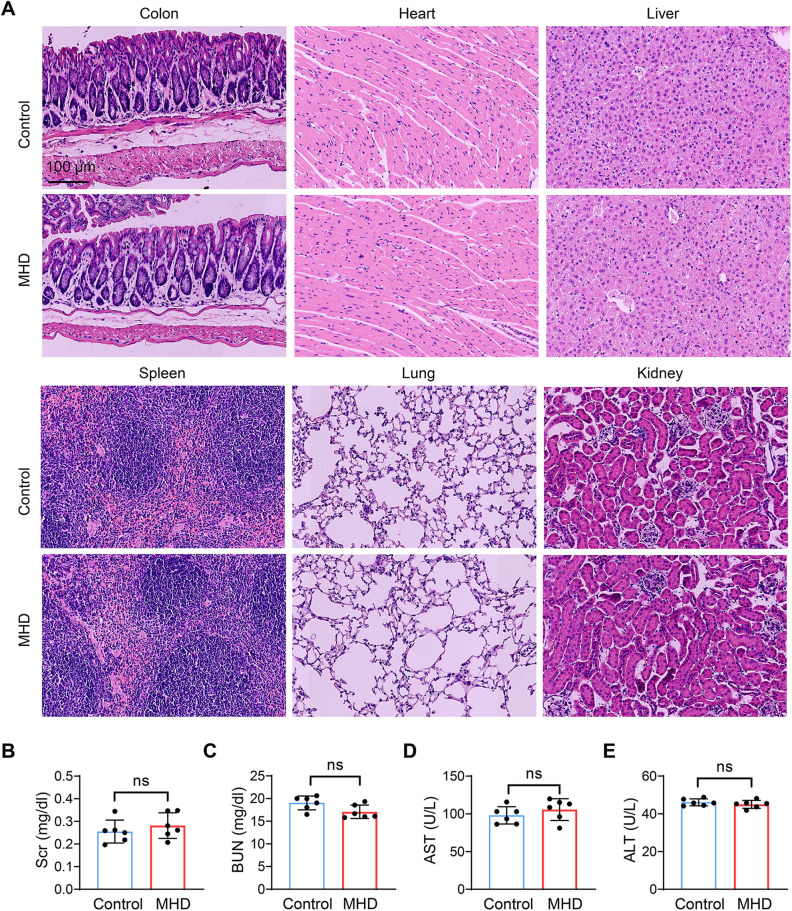
In vivo safety assessment of MHD. Mice were treated with MHD (50 mg/kg) for 11 days, and then the mice were sacrificed for HE staining (A), Scale bar = 100 μm. (B-E) Blood samples were collected to perform hepatic and renal function tests, Scr, creatinine; BUN, blood urea nitrogen; ALT, alanine transaminase; AST, aspartate transaminase. n = 6. ns, not significant.

To assess the protective role of MHD in UC, DSS-induced UC mice were treated with different doses of MHD or SASP as a positive control. Surprisingly, MHD treatment significantly alleviated DSS-induced colonic shortening, as well as symptoms of diarrhea and bloody stools, with effects comparable to those observed in the SASP-treated group (Fig 8A). Mice receiving MHD exhibited significantly less weight loss compared to the UC group, and this protective effect was also evident in the SASP group (Fig 8B). Furthermore, histopathological analysis revealed that both MHD and SASP administration effectively mitigated colonic epithelial cell desquamation, inflammatory cell infiltration, and extensive edema in the muscle layer (Fig 8C). Similarly, we found that MHD significantly reduced DSS-induced up-regulation of TNF-α, IL-6 and SLC7A11 while increasing the SLC26A2 expression in UC mice, similar to the effect observed with SASP treatment (Fig 8D–G). Moreover, we evaluated the safety of MHD. Histological examination of vital organs such as colon, heart, liver, spleen, lung and kidney showed no pathological damage in MHD-treated mice (Fig 9A). In addition, there was no significant difference in liver and kidney functions between control and MHD-treated mice (Fig 9B–D). These results indicate that MHD has a significant therapeutic effect on UC and has promising biosafety.

## Discussion

This study presents the first comprehensive analysis of DRG expression in colon tissues from UC and non-UC patients. By examining the relationship between disulfidptosis and UC, new and important insights into the pathogenesis of UC were revealed. We found that SLC7A11, an essential gene associated with disulfidptosis, was significantly induced in UC. The protein encoded by SLC7A11, known as xCT subunit, is an amino acid transporter which is involved in the intracellular transport of cystine and glutathione [13]. Additionally, SLC7A11 exerted a critical function in disulfidptosis, where its elevated expression causes irregular disulfide accumulation, consequently inducing the collapse of cellular proteins and cytoskeleton, ultimately initiating disulfide-triggered cell death [10]. It has been shown that SLC7A11 expression is significantly upregulated in UC patients [36]. One study suggested that inhibiting the activation of the miR-144-3p/SLC7A11 signaling pathway could alleviate DSS-induced colitis in mice [37]. These findings suggest that disulfidptosis, as a novel form of cell death, may be associated with UC [37]. Furthermore, our findings also highlighted differences in immune cell composition in UC patients, with a significant increase in infiltrating macrophages, activated mast cells, resting NK cells, and resting CD4 memory T cells. Immune cells are pivotal in therapeutic strategies for UC. For example, T cell immunotherapy is a key approach that modulates immune cell activity to reduce inflammation and control disease progression [38]. In addition, mast cells played a key role in the treatment of UC, especially in resisting bacterial infections and inflammation [39].

This study further explored the expression patterns of DRGs in UC at the molecular level. Interestingly, a cluster with elevated expression of SLC7A11, SLC3A2, MYH10, FLNA, RPN1, and PRDX1 correlated with a more severe damage phenotype. From the DRGs cluster, we identified 69 DEGs and two key modules. GO analysis showed that the DEGs were linked to processes like leukocyte migration, cytokine activity, and immune response. UC is an immune-mediated disorder of the digestive system, where immune cells regulate disease progression through their infiltration into affected areas. Additionally, KEGG pathway analysis further showed that the DEGs were enriched in pathways like cytokine-cytokine receptor interaction, NF-kappa B signaling, AGE-RAGE signaling in diabetic complications, and tryptophan metabolism. Our findings aligned with comparing UC patients with those receiving treatment or healthy individuals, showing that inhibiting the Nf-kB and TNF-α signaling pathways can effectively prevent and alleviate UC symptoms [40–42]. Diabetes is a common complication among UC patients [43]. A recent large-scale case-control study revealed that, among more than 1,200 children diagnosed with inflammatory bowel disease (IBD), including 488 with UC, the diabetes prevalence was higher in the UC patients compared to the control patients [44]. Furthermore, UC is a complex systemic inflammatory immune response caused by multiple factors [45], and similar to other immune-related diseases, UC shows a strong association with diabetes in both children and adults [43]. Our study suggests that the AGE-RAGE signaling pathway could be a potential therapeutic target for diabetes-related UC treatment.

Through the application of machine learning algorithms, we identified hub genes associated with disulfidptosis, namely CXCL1, HMGCS2, AQP8, and SLC26A2. Notably, CXCL1 encodes a chemokine, alternatively referred to as GRO-α, which orchestrates the attraction of white blood cells to gather at inflammatory sites during inflammation [46]. In cases of inflammatory bowel diseases, the heightened expression of CXCL1 could potentially intensify disease progression and worsen symptoms by fostering the accumulation of inflammatory cells and promoting white blood cell adhesion [47,48]. HMGCS2, a pivotal enzyme in the glycerolipid synthesis pathway, has been identified by Kim and colleagues as capable of attenuating TNFα-induced intestinal cell apoptosis through its overexpression [49]. AQP8 participates in maintaining the balance of intracellular and extracellular water. In UC, the atypical expression of AQP8 could potentially relate to disrupted intestinal mucosal water balance and inflammation [50]. SLC26A2 encodes a sulfate-chloride co-transporter protein involved in the transport of sulfate and chloride ions, playing a pivotal role in cartilage development [51]. Studies have shown that mutations or pathogenic variants of SLC26A2 can lead to impaired sulfate absorption in intestinal epithelial and chondrocyte cells, thereby disrupting extracellular matrix homeostasis [52]. In addition, SLC7A11 is a key regulator of Disulfidptosis, and its mediated cystine uptake provides a substrate for the synthesis of sulfur-containing antioxidants [53], whereas SLC26A2 is involved in the synthesis of glycosaminoglycans by supplying sulfate [54]. This suggests that SLC26A2 and SLC7A11 may synergistically regulate Disulfidptosis during UC. Moreover, our data indicate that SLC26A2 downregulation may exacerbate UC-related tissue damage by amplifying the inflammatory response through disulfide accumulation. Although the direct interaction of SLC26A2 and SLC7A11 in the UC process has not been fully revealed, these results suggest that they may have potential synergistic regulatory mechanisms in the inflammatory microenvironment. Additionally, we identified MHD, a key component of Pueraria Mirifica in traditional Chinese medicine, which inhibited LPS-induced inflammatory responses in Caco2 cells and alleviated DSS-induced colonic injury in UC mice by upregulating SLC26A2. These results imply that targeting SLC26A2 may offer a potential therapeutic approach for UC.

## Conclusion

In conclusion, this study offers new perspectives on the pathogenesis of UC, particularly the role of disulfidptosis. These findings have important clinical implications for deepening our understanding of UC, uncovering new therapeutic targets, and advancing personalized treatment strategies. However, further studies are needed to confirm these findings and investigate the complex relationship between disulfidptosis and UC, to provide better clinical guidance.

## Supporting information

S1 Table Characterization of disulfidptosis-related genes. (XLSX)

S2 Table Screening of Traditional Chinese Medicines. (XLSX)

S3 Table Effective components of Radix Puerariae. (XLSX)

S4 Table The primer sets for qPCR. (XLSX)

S5 Table DEGs of UC vs control. (XLSX)

S6 Table Clinical information for GSE73661. (XLSX)

S7 Table Clinical information for GSE92415. (XLSX)

S8 Table Clinical information for GSE73661 and GSE92415 merged. (XLSX)

S9 Table Statistical analysis of clinical information for GSE73661 and GSE92415 merged. (XLSX)

S1 Fig The flow-process diagram. (TIF)

S2 Fig Expression of key DRGs in DSS-induced UC mice. (TIF)

S3 Fig Expression of hub genes in different DRG clusters. (TIF)

S4 Fig Validation of hub genes based on external datasets. (TIF)

S1 Raw Images Original uncropped images for Western blot. (PDF)
