# Supplementary figures and images for: Screening and validation of 3’-Methoxydaidzein as a therapeutic agent in ulcerative colitis based on disulfidptosis-associated molecular clusters
Source: PLoS One. 2025 Jun 6;20(6):e0324586. doi: 10.1371/journal.pone.0324586 (PMC12143574; doi:10.1371/journal.pone.0324586)

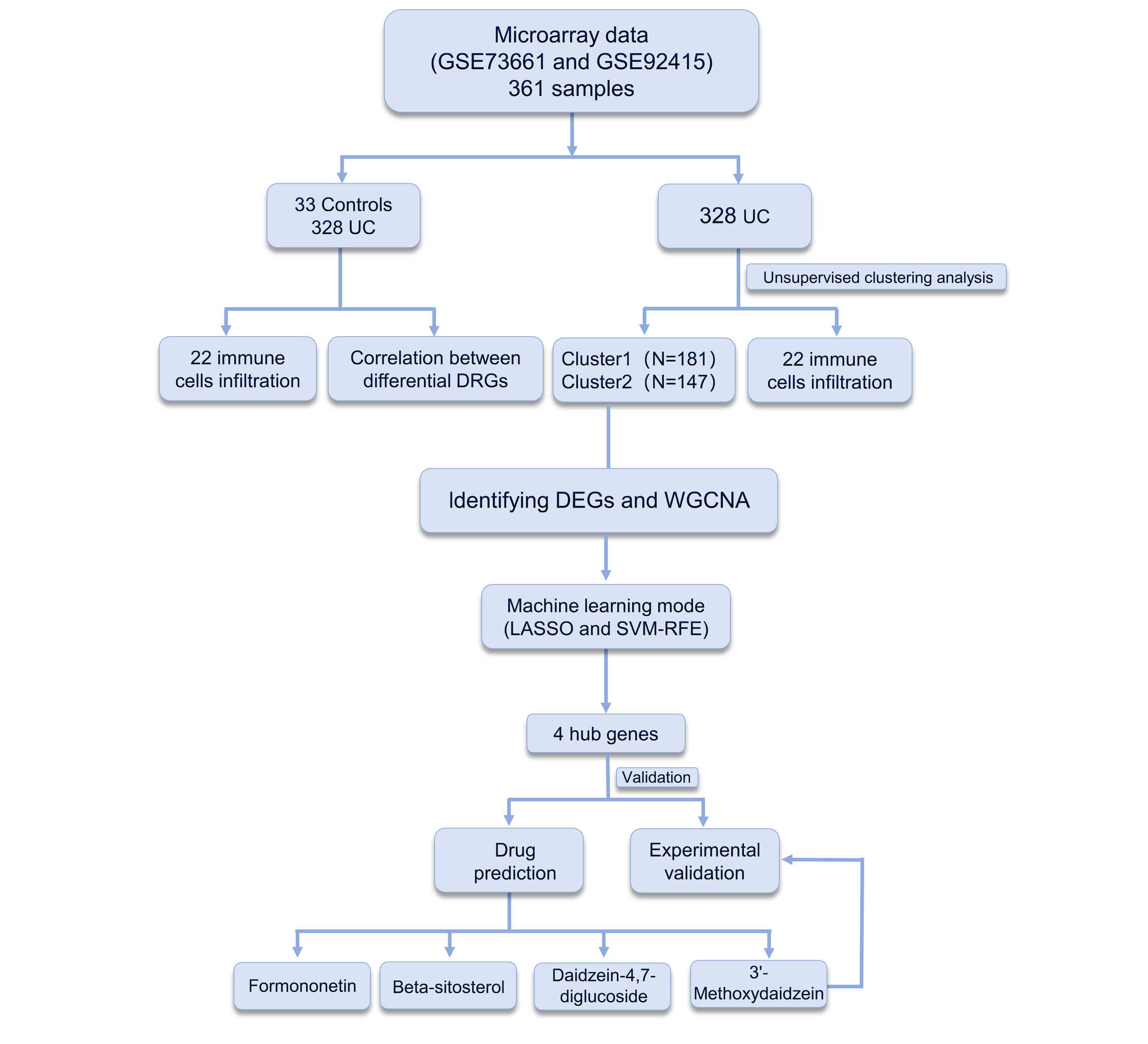

Supplement: S1 Fig — (TIF) [file pone.0324586.s010.tif]

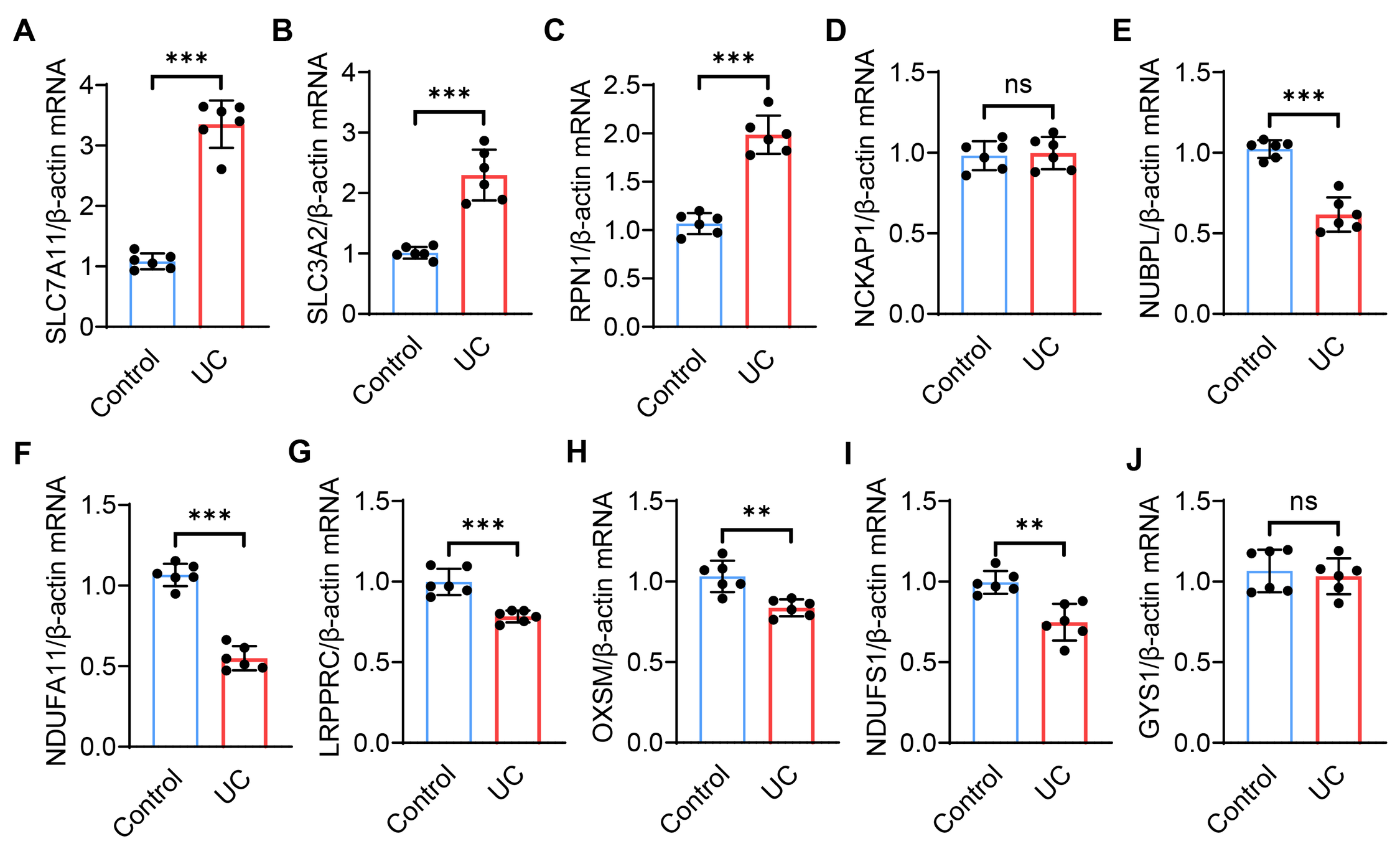

Supplement: S2 Fig — (TIF) [file pone.0324586.s011.tif]

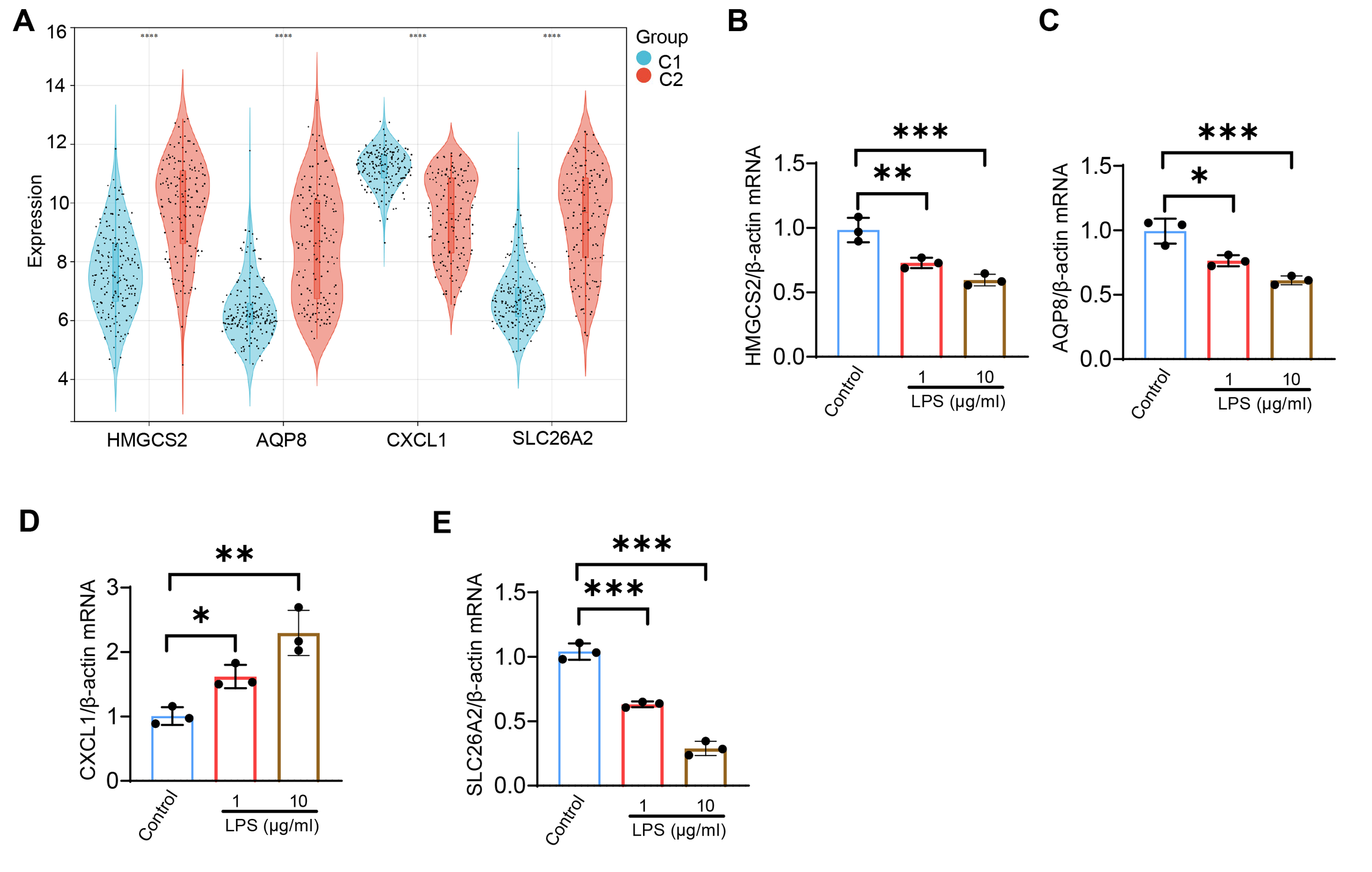

Supplement: S3 Fig — (TIF) [file pone.0324586.s012.tif]

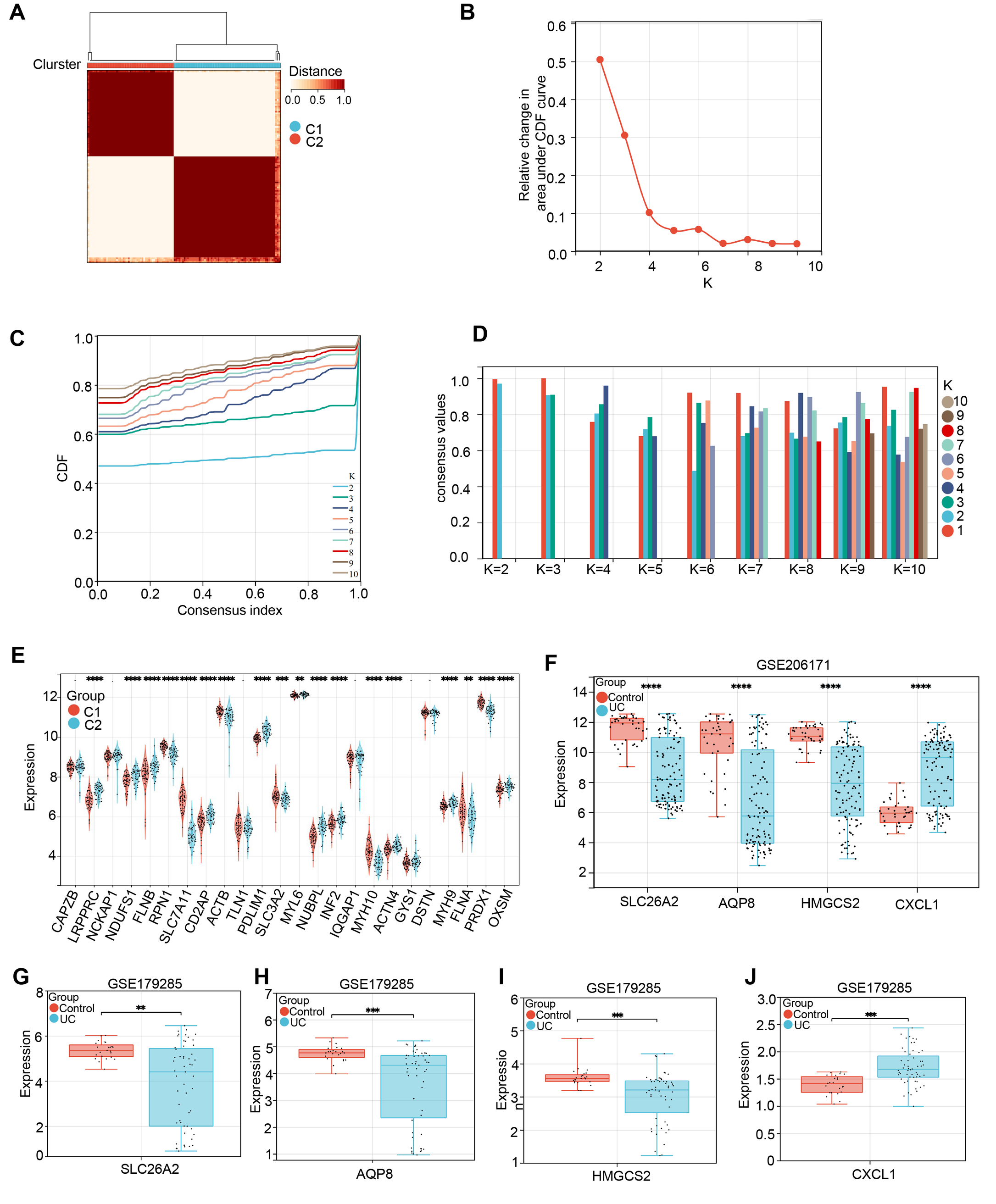

Supplement: S4 Fig — (TIF) [file pone.0324586.s013.tif]
