# Supplementary material for: Screening and validation of 3’-Methoxydaidzein as a therapeutic agent in ulcerative colitis based on disulfidptosis-associated molecular clusters
Source: PLoS One. 2025 Jun 6;20(6):e0324586. doi: 10.1371/journal.pone.0324586 (PMC12143574; doi:10.1371/journal.pone.0324586)
Supplement: S1 Raw Images — (PDF) [file pone.0324586.s014.pdf]

Figure 5G

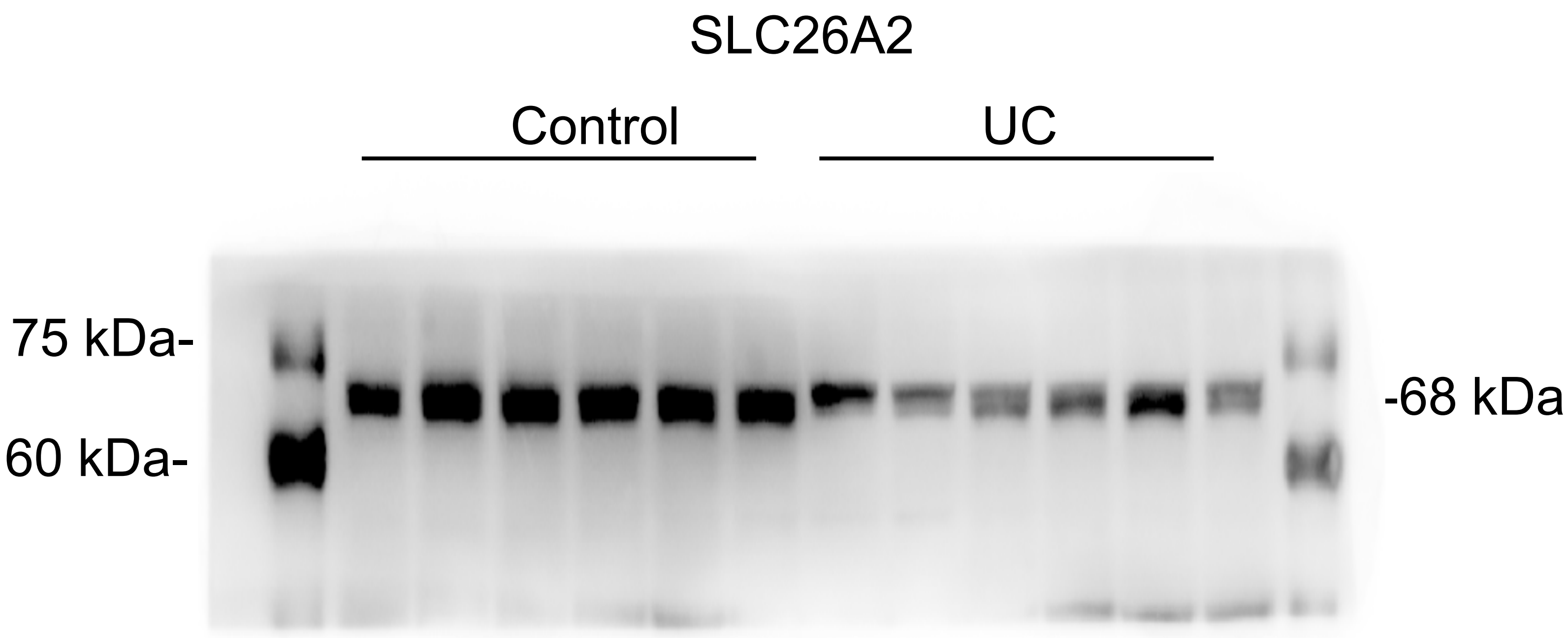

Figure 5G

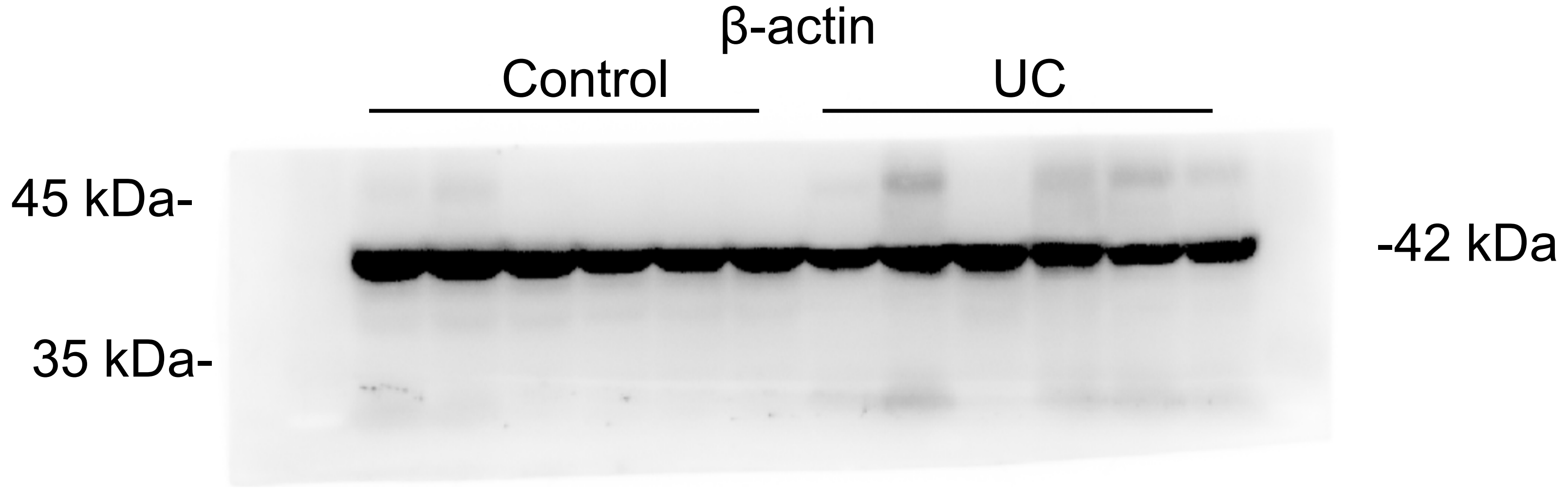

Figure 7L

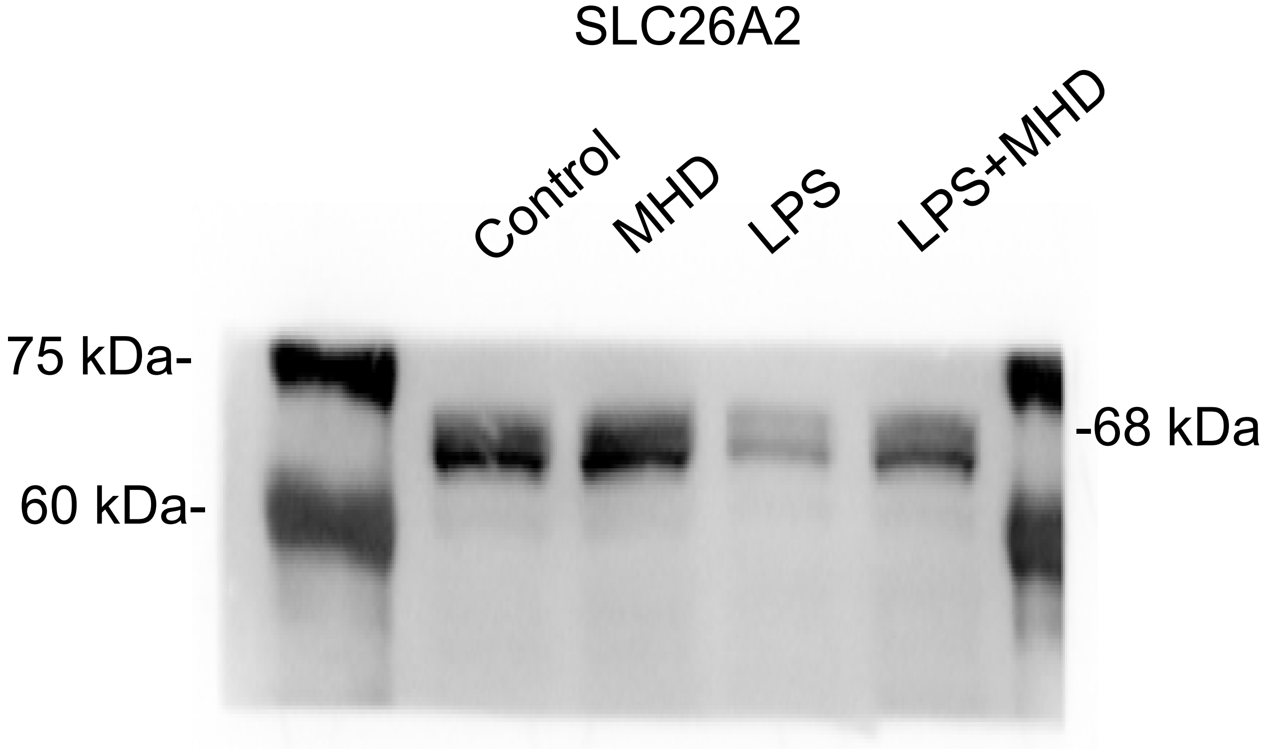

Figure 7L

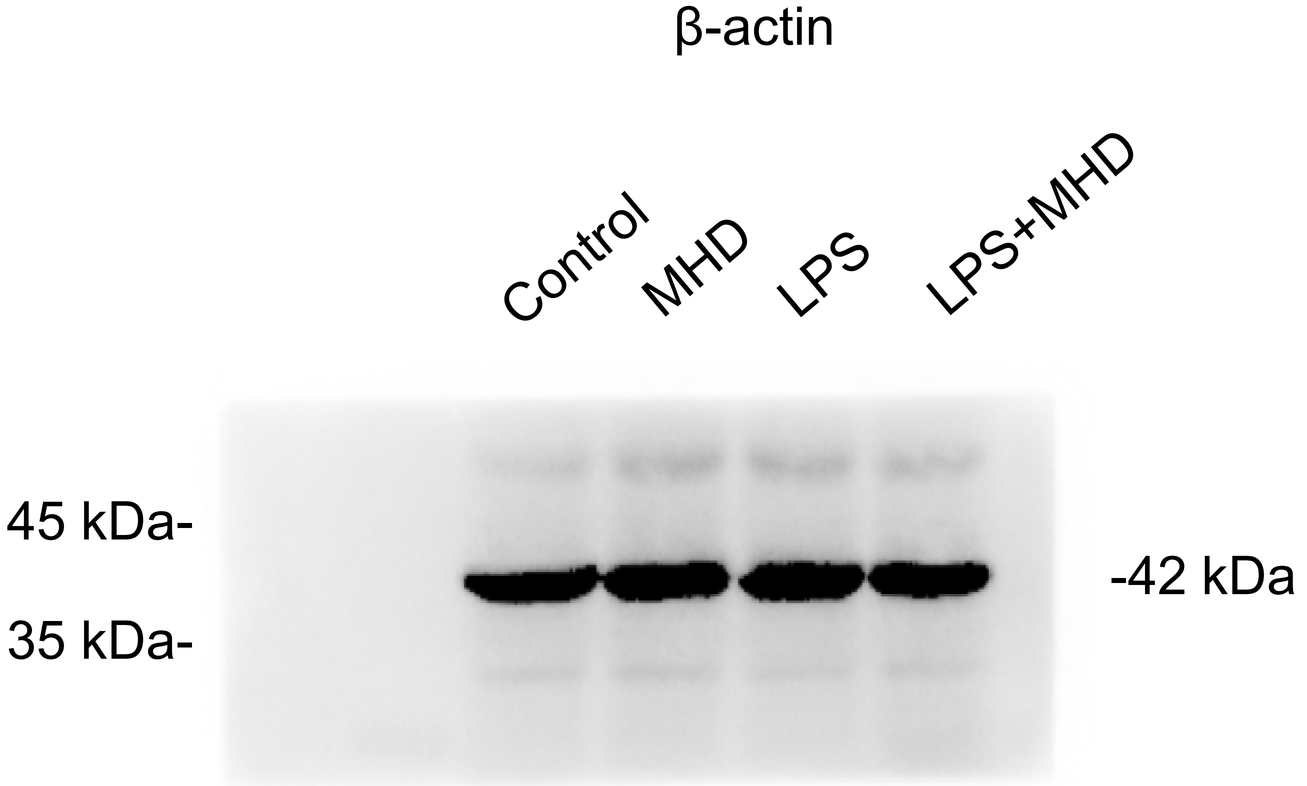

Figure 7M

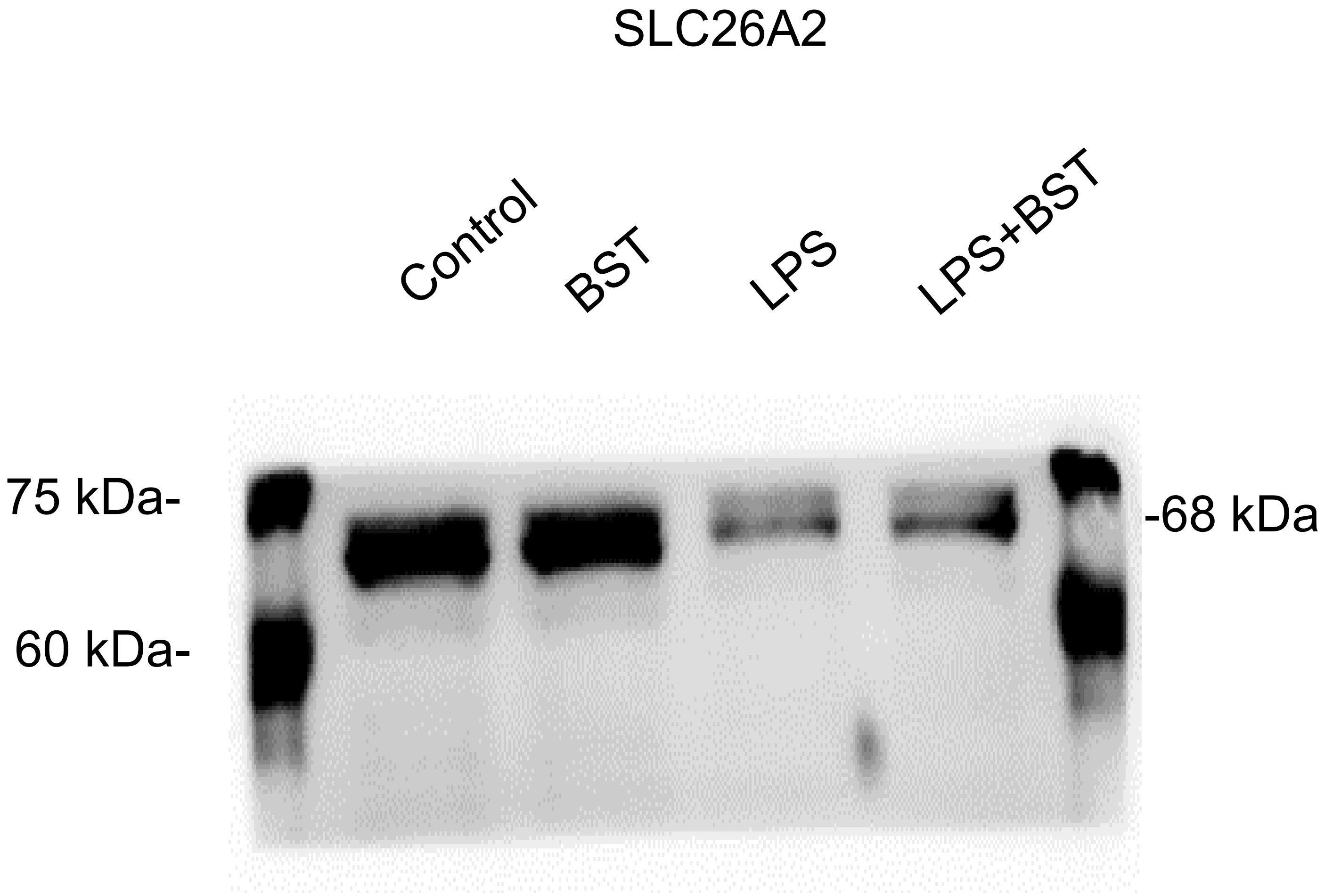

Figure 7M

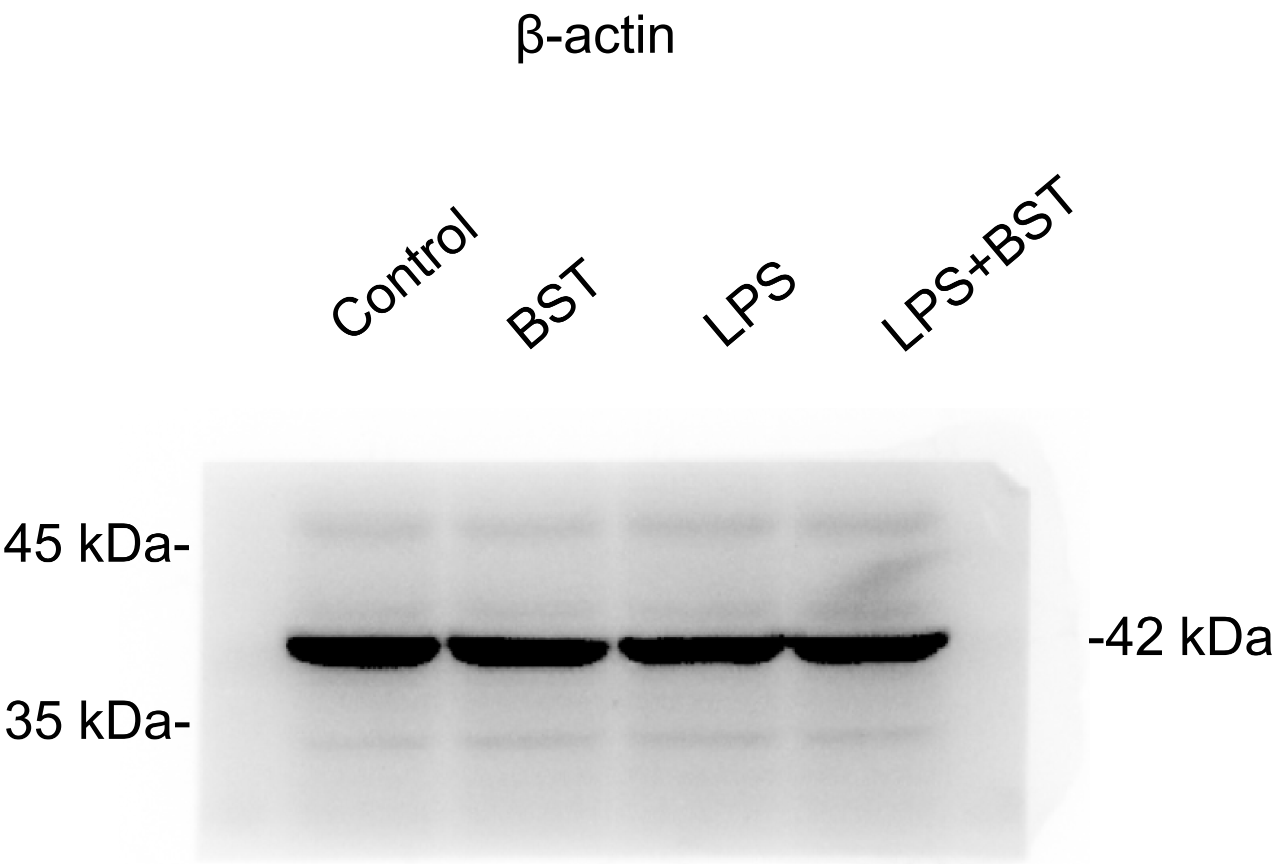

Figure 7N

SLC26A2

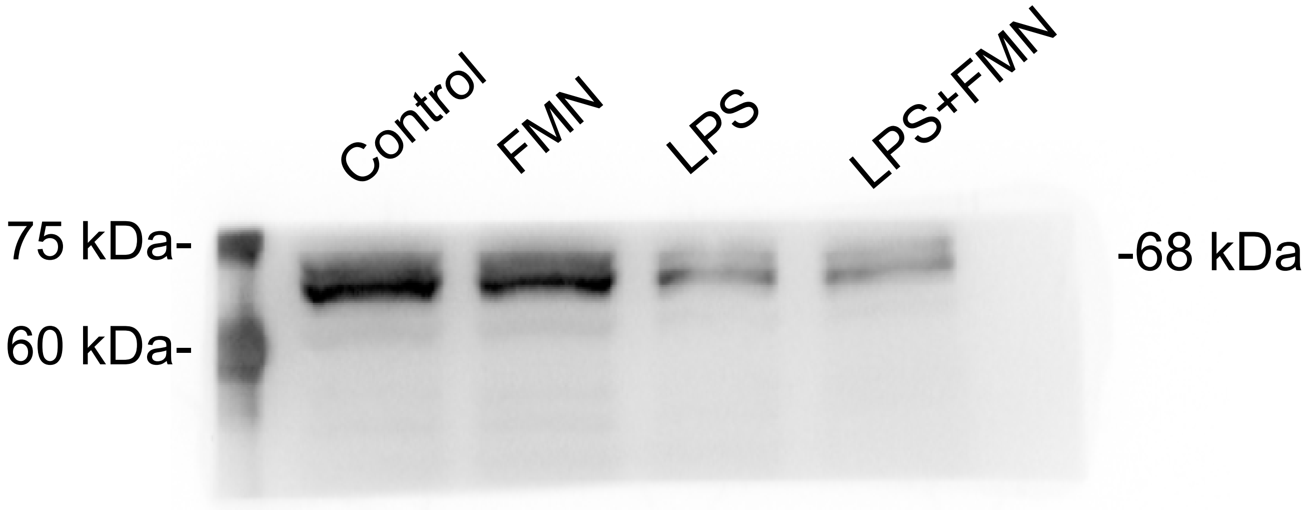

Figure 7N

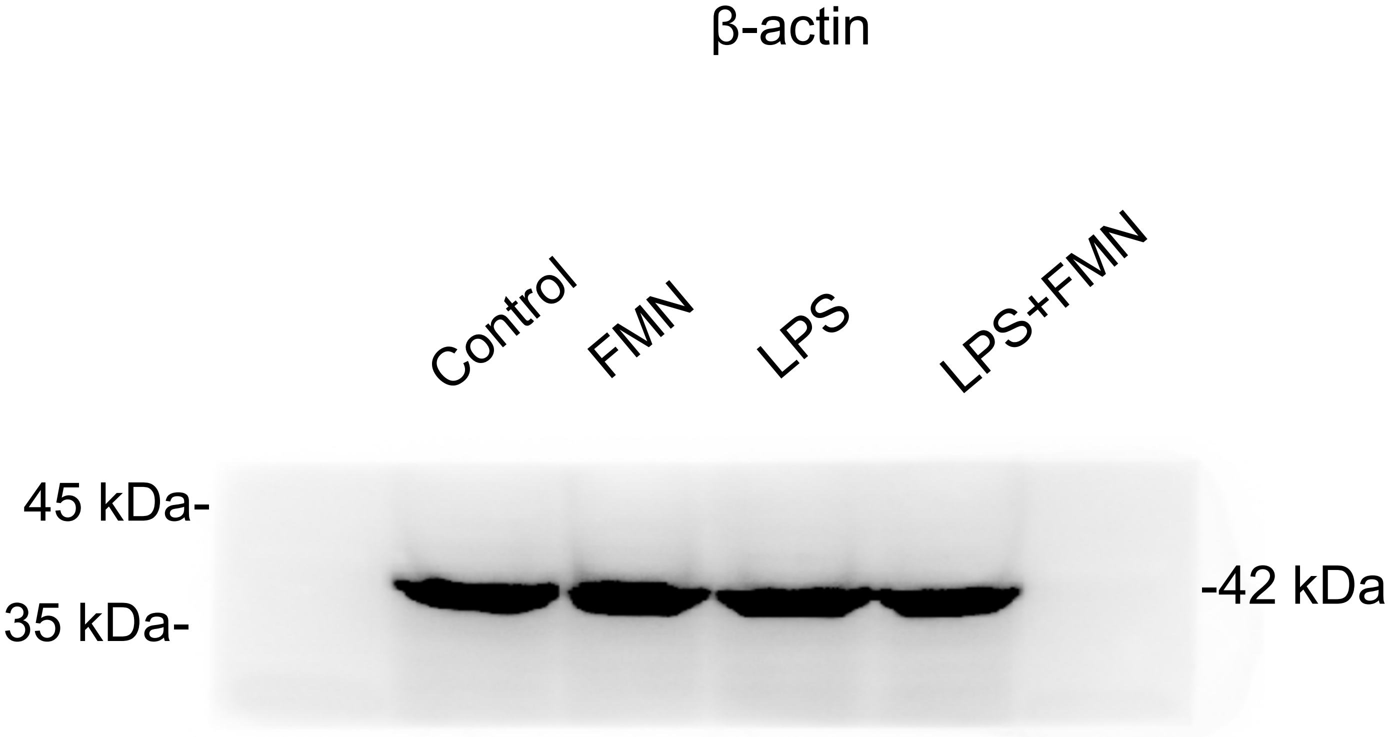

Figure 7O

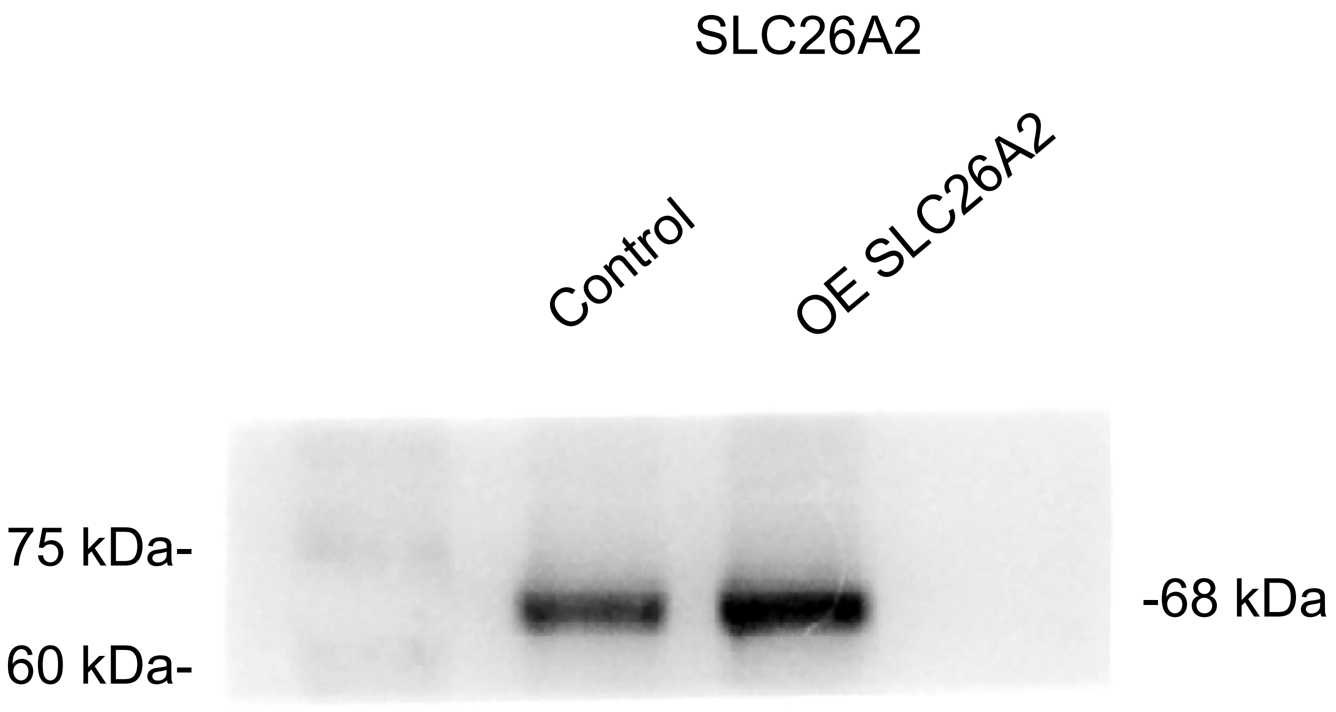

Figure 7O

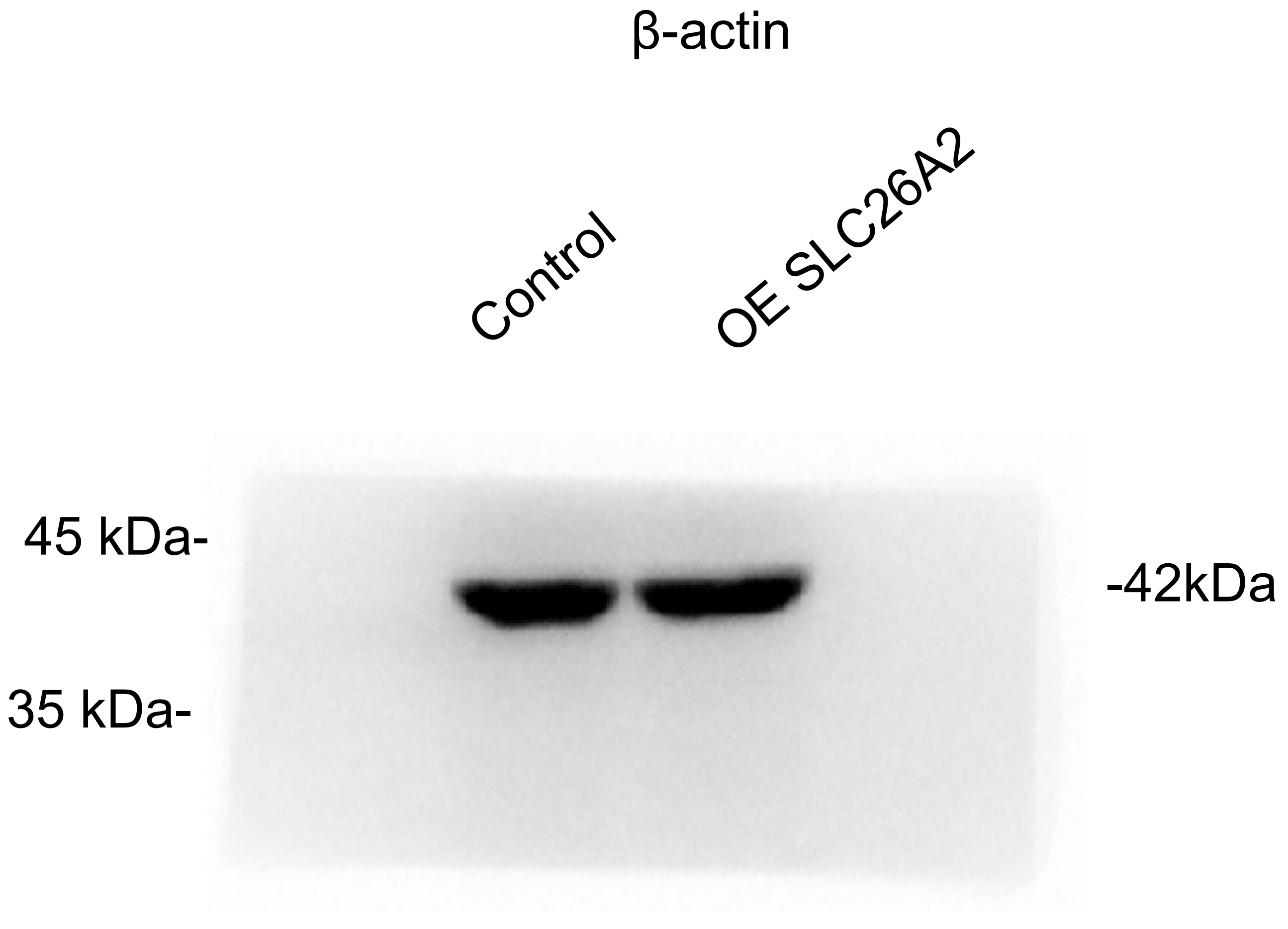

Figure 8F

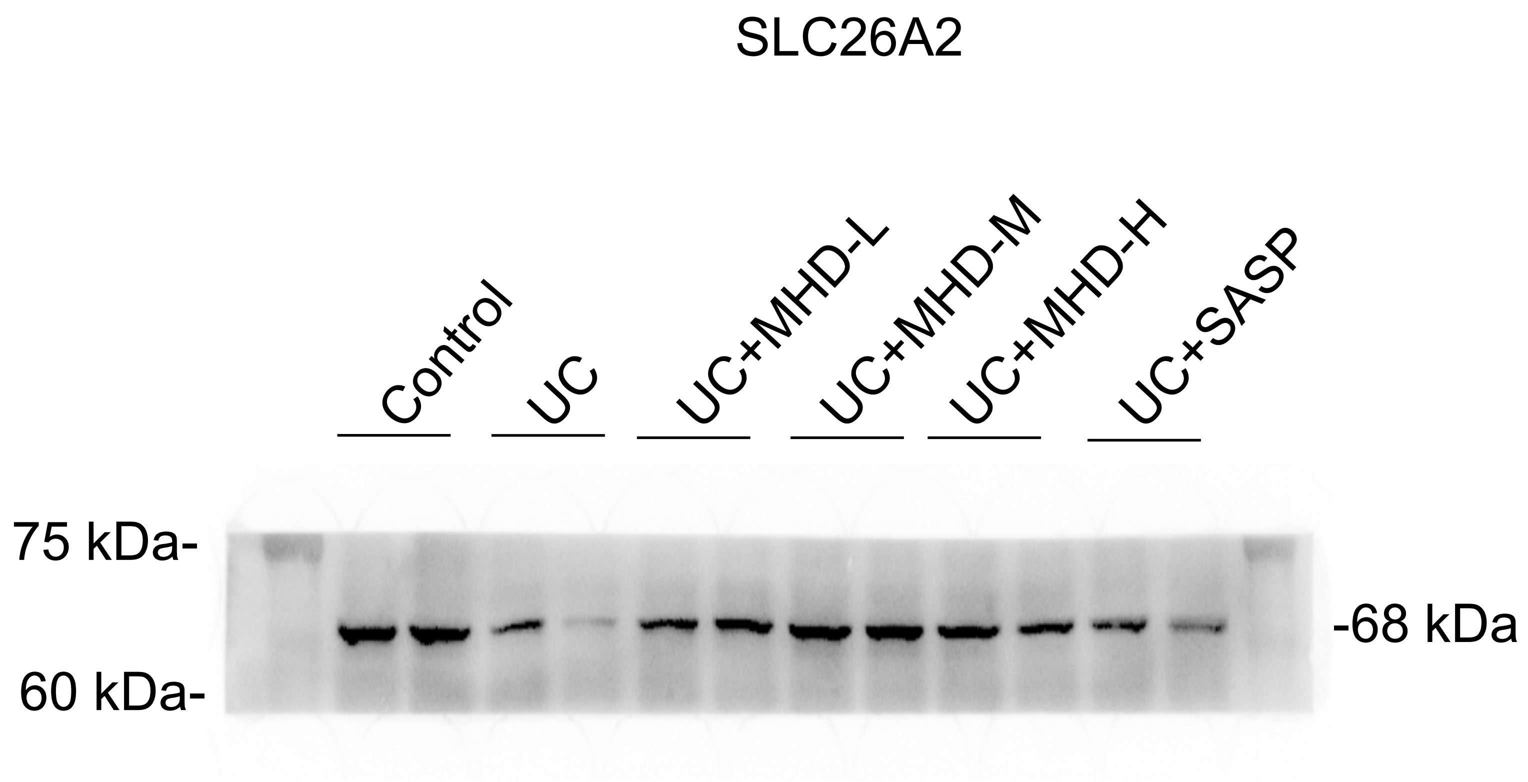
SLC26A2
Control
UC
UC+MHD-L
UC+MHD-M
UC+MHD-H
UC+SASP
75 kDa-
60 kDa-
-68 kDa

Figure 8F

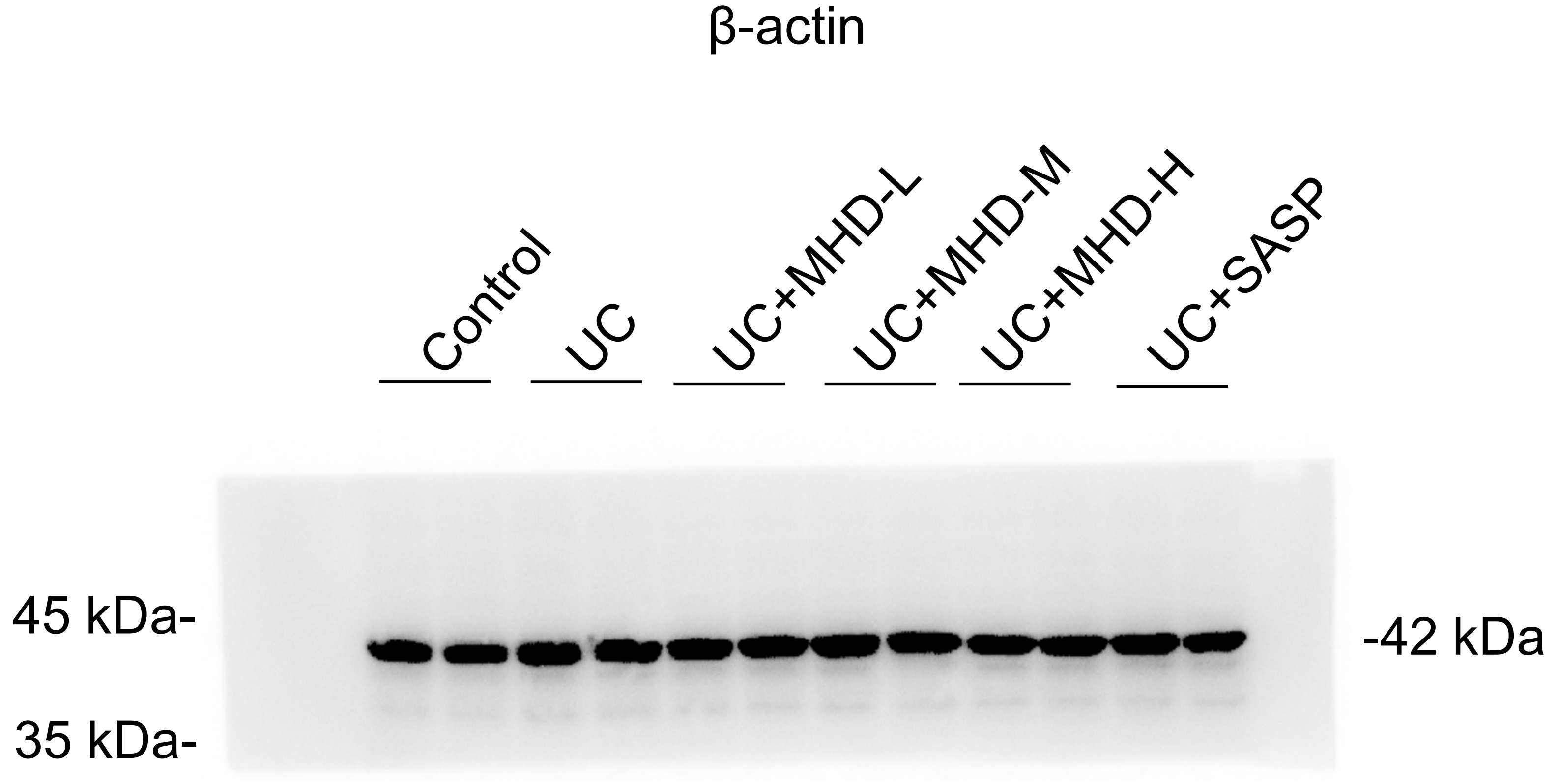
β-actin
Control
UC
UC+MHD-L
UC+MHD-M
UC+MHD-H
UC+SASP
45 kDa-
35 kDa-
-42 kDa
